# Experimental and Theoretical Study of Defect Evolution in InSb Epilayers under Gamma Irradiation: A Comparative Analysis of MOCVD vs MBE Growth Methods

**DOI:** 10.1021/acsomega.5c10490

**Published:** 2025-12-15

**Authors:** John Fredy Ricardo Marroquin, Alex Cortes Derc, Erika Nascimento Lima, Igor Saulo Santos de Oliveira, Mustafa Gunes, Mustafa Akyol, Braulio S. Archanjo, Walter M. de Azevedo, Mohamed Henini, Jorlandio Francisco Felix

**Affiliations:** † LabINS, Institute of Physics, 28127 University of Brasília (UnB), Federal District, Brasília 70910-900, Brazil; †† UnB Planaltina Faculty, University of Brasília, Brasília-DF 70904-910, Brazil; ‡ 67826 Instituto de Física, Universidade Federal de Mato Grosso, Cuiabá, Mato Grosso 78060-900, Brazil; § Departamento de Física, 67739 Universidade Federal de Lavras, C.P. 3037, Lavras, Minas Gerais 37203-202, Brazil; ∥ Department of Materials Science and Engineering, 365074 Adana Alparslan Türkeş Science and Technology University, Adana TR-01250, Turkey; ⊥ Materials Metrology Division, National Institute of Metrology, Quality and Technology (INMETRO), Duque de Caxias, Rio de Janeiro 25250-020, Brazil; # Departamento de Química Fundamental, 28116 Universidade Federal de Pernambuco, Recife, Pernambuco 50740-560, Brazil; ∇ School of Physics and Astronomy, Nottingham Nanotechnology and Nanoscience Center, 6123 University of Nottingham, Nottingham NG7 2RD, U.K.

## Abstract

The operational requirements of high-radiation and extraterrestrial environments highlight the need to evaluate narrow-bandgap semiconductors that remain unexplored under such conditions, among them Indium Antimonide (InSb). As a material system, InSb offers unparalleled electron mobility and a massive *g*-factor, making it indispensable for next-generation infrared detection, Hall sensing, and topological quantum computing architectures. However, the practical realization of these devices is frequently hindered by the necessity of heteroepitaxial growth on lattice-mismatched substrates, typically Gallium Arsenide (GaAs), which introduces a complex landscape of threading dislocations and interfacial defects. This report presents an exhaustive, multimodal investigation into the radiation hardness of InSb epilayers, specifically contrasting the microstructural evolution of films grown via Metal–Organic Chemical Vapor Deposition (MOCVD) against those synthesized by Molecular Beam Epitaxy (MBE). Utilizing an experimental framework that integrates Electron Paramagnetic Resonance (EPR), Raman spectroscopy, High-Resolution Scanning Transmission Electron Microscopy (HR-STEM), and ab initio Density Functional Theory (DFT), this study elucidates the mechanistic divergence in radiation response between the two growth methodologies. The data reveal a critical, counterintuitive trade-off: the MOCVD-grown material, despite exhibiting superior initial crystalline quality driven by a zinc-doped seed layer that passivates interfacial traps, demonstrates a heightened susceptibility to electronic degradation and stoichiometry violation under high-fluence Gamma (γ) irradiation. In contrast, the MBE-grown material, initially marred by a higher density of dislocations, exhibits a complex “survivability” mode at elevated doses, characterized by defect saturation. This report details the atomic-level physics driving these behaviors, including the radiation-induced formation of homopolar Sb–Sb bonds, the symmetry-breaking anisotropy of the *g*-factor, and the thermodynamic instability of dopant-passivated interfaces under nonequilibrium conditions. Furthermore, these findings can be used as actionable engineering guidelines for Radiation Hardness Assurance (RHA), proposing novel nondestructive spectroscopic metrics for the qualification of semiconductors destined for space and nuclear applications.

## Graphical abstract

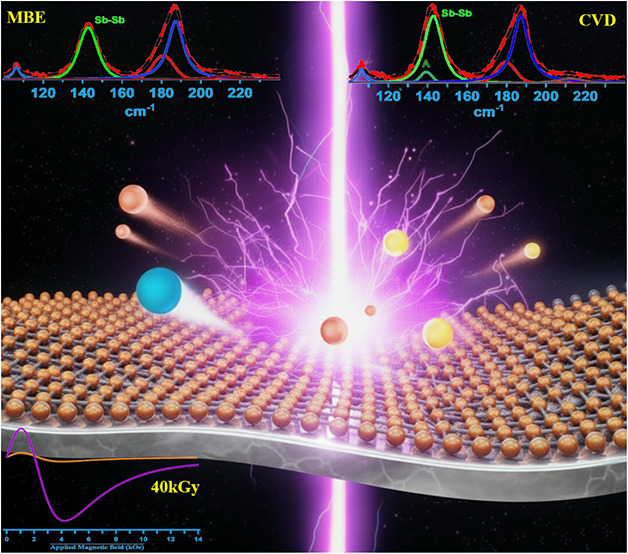

## Introduction

Among the III–V binary compounds, InSb occupies a distinct position at the extreme end of the property spectrum. Crystallizing in the zinc-blende structure, InSb is characterized by the narrowest direct bandgap (*E*_*g*_ ≈ 0.17 eV at 300 K and 0.23 eV at 80 K) and the smallest electron effective mass $\left(m_{e}^{*}\approx 0.014m_{0}\right)$ of any conventional semiconductor.^1–3^ These fundamental electronic parameters govern its macroscopic behavior, resulting in high room-temperature electron mobility that can exceed 77,000 cm^2^/V·s in high-purity bulk crystals, and potentially reach 200,000 cm^2^/V·s under cryogenic conditions.^4^ This extreme mobility makes InSb the material of choice for high-speed electronic devices, extremely sensitive Hall effect sensors, and magnetoresistors.^5–11^ Furthermore, the narrow bandgap corresponds directly to photon absorption in the 3–5 μm mid-infrared (MWIR) spectral window, rendering InSb the preeminent material for thermal imaging focal plane arrays (FPAs) used in aerospace, defense, and astronomical applications.^12^

Beyond classical optoelectronics, InSb has emerged as a central platform for quantum information science. The material exhibits a giant Landé *g*-factor (|*g*| ≈ 51 for bulk) and strong spin–orbit coupling (SOI).^13^ These properties are prerequisites for the manipulation of electron spins in spintronic devices and for the realization of Majorana zero modes in semiconductor, superconductor hybrid nanowires, quasiparticles that form the basis of topologically protected quantum computing.^14^ The integrity of these quantum states is fundamentally linked to the crystalline perfection of the host lattice; thus, understanding defect dynamics is not merely a materials engineering concern but also a necessity for quantum coherence.

Despite its superlative electronic properties, the wide-scale deployment of InSb is constrained by the lack of lattice-matched, semi-insulating substrates. Bulk InSb substrates are conductive, brittle, and available only in small diameters compared with the industry-standard GaAs or Silicon wafers. Consequently, InSb device layers are typically synthesized via heteroepitaxy on GaAs substrates. This integration presents a formidable thermodynamic challenge, the lattice constant of InSb (6.479 Å) is approximately 14.6% larger than that of GaAs (5.653 Å).^15,16^ In the context of epitaxial growth, a mismatch of this magnitude is immense. According to the Frank-van der Merwe model of crystal growth, strain energy accumulates in the depositing layer until a critical thickness is reached, beyond which the strain is relaxed through the formation of misfit dislocations at the interface.^17,18^ Given the 14.6% mismatch, the critical thickness for coherent growth in InSb/GaAs is less than a single monolayer.^15^ This forces the growth mode into the Volmer–Weber (island growth) regime almost immediately.^19^ As these 3D islands coalesce, misalignment of their crystal lattices results in a high density of threading dislocations (TDs) that propagate vertically from the interface into the active device layer. Typical threading dislocation densities for InSb on GaAs can range from 10^8^ to 10^9^ cm^–2^ unless sophisticated buffer layer strategies are employed. These dislocations are not electrically inert. They create deep-level acceptor states within the bandgap, acting as potent scattering centers that degrade carrier mobility and as nonradiative recombination centers that quench optical emission and detection efficiency.^17,18^ The reduction of this defect density is the primary objective of epitaxial optimization, driving competition between growth techniques such as MBE and MOCVD.^20–25^

The deployment of InSb devices in extraterrestrial environments exposes them to a spectrum of ionizing radiation that is absent on the Earth’s surface. Satellites in Low Earth Orbit (LEO) traverse the Van Allen radiation belts, encountering high fluxes of trapped protons and electrons.^26^ Deep space missions face galactic cosmic rays (heavy ions) and solar particle events (protons and heavy ions) and γ rays.^27^ Nuclear reactor monitoring systems are subjected to neutron fluxes and intense γ radiation fields.^28^ In this regard, a detailed understanding of how different types of radiation interact with semiconductor lattices is essential for predicting and mitigating radiation-induced damage in InSb devices and related materials.

From a microscopic perspective, the interaction of high-energy radiation with a semiconductor lattice is a complex process that results in structural defects and the subsequent modification of material properties. The specific mechanism of defect formation depends critically on the type of incident particle or photon. Energetic particles, such as neutrons and protons, are particularly effective at inducing displacement damage.^29^ This phenomenon arises when an incident particle transfers substantial kinetic energy to a lattice atom, displacing it from its equilibrium position. This displacement generates a vacancy-interstitial pair commonly known as a Frenkel defect. In silicon, for example, a high-energy proton or neutron (≈1 MeV) can trigger a collision cascade, leading to complex defect clusters that can extend for hundreds of angstroms within the crystal lattice.^30^ In the context of InSb-based Hall sensors, it has been demonstrated that a high initial electron concentration is essential for maintaining a stable electrical response in environments dominated by thermal and epithermal neutrons.^31^ This requirement highlights the material’s resilience to such damage, which can otherwise degrade device performance.

Furthermore, while materials such as GaP, GaAs, and Ge exhibit resilience against swift heavy ions, manifesting only discontinuous defect tracks, InSb and InP are highly susceptible to the formation of continuous amorphous tracks. These tracks progressively overlap with increasing ion fluence, eventually yielding a fully amorphous layer.^32^ This disparity is rationalized by the thermal spike model, which postulates that amorphous damage manifests when the deposited electronic energy surpasses a critical threshold, inducing localized lattice melting. Furthermore, established research indicates that the presence of preexisting point defects and clusters significantly enhances the efficiency of electron–phonon coupling.^32^ This finding is particularly relevant for thin films grown on lattice-mismatched substrates such as InSb on GaAs, which are known to possess an initially higher defect density. It therefore suggests that these materials will experience a more efficient energy transfer from the electronic subsystem to the lattice, leading to more pronounced radiation damage.

We specifically focus on the effects of Gamma (γ) irradiation. While heavy ions cause dense ionization tracks and massive displacement cascades, γ rays represent a pervasive, penetrating threat that degrades materials through cumulative dose effects (Total Ionizing Dose, TID) and displacement damage caused by secondary electrons. On a microscopic scale, ionizing radiation, such as high-energy γ rays, interacts primarily with the material’s electronic system. Although these species are capable of inducing displacement damage, the effective cross-section for direct momentum transfer to atomic nuclei is significantly lower than that of heavier particles. Consequently, gamma irradiation tends to predominantly generate isolated point defects rather than extended defect clusters. For narrow-bandgap materials like InSb, a particularly important distinction is that defects can also be generated through an indirect ionization process.^33^ In this mechanism, soft X-rays or γ rays produce secondary photoelectrons with energies well below the threshold for direct collision-based displacement. The displacement of lattice atoms is then initiated by these photoelectrons, a process that has been likened to the Varley mechanism observed in ionic crystals.^33^ This fundamental difference in the dominant generation pathway explains why the defect population from gamma irradiation is expected to be distinct from that produced by heavy-particle bombardment.

The introduction of lattice defects, whether as isolated point defects or as complex clusters, profoundly influences the electrical and optical properties of a semiconductor. Vacancies, interstitials, and their associated complexes introduce new energy levels within the material’s band gap. These defect states act as efficient scattering and recombination centers for charge carriers, resulting in significant alterations of observable material properties. For instance, in silicon carbide (SiC) MOSFETs, low-fluence gamma irradiation initially induces interface traps at the SiC/SiO_2_ interface, which can counterintuitively cause an initial increase in drain current. However, with increasing cumulative dose, the progressive generation of bulk lattice defects results in a marked decline in drain current and a substantial increase in gate leakage.^30^ While these degradation pathways in materials like SiC are well documented, the atomic-level defect mechanisms and their correlation with the growth method (MOCVD vs MBE) response of InSb to high-energy radiation, particularly γ rays, remain uninvestigated.

To bridge these interconnected gaps, we present a systematic investigation of the structural and electronic properties of InSb epilayers grown by MBE and MOCVD on GaAs (001) substrates. Using a combination of EPR and Raman spectroscopy, complemented by Density Functional Theory (DFT) calculations, we establish a quantitative correlation between the growth technique, the resulting defect landscape, and the material’s response to varying gamma-ray doses. This study provides crucial insights into the fundamental mechanisms of radiation-induced damage in InSb and offers a pathway for developing radiation-hardened devices for extreme environments.

## Experimental Detail

InSb epilayers were grown on GaAs (001) substrates via MBE and MOCVD systems by utilizing a GaAs buffer layer. Detailed growth procedures are described in ref. 34. Briefly, the InSb film grown by the MBE system (hereafter InSb-MB) employed a 100 nm InSb seed layer deposited on GaAs (001) with a temperature ramp from 330 °C to 400 °C over 15 min, followed by the growth of a 1 μm InSb layer at 500 °C. In contrast, the InSb film grown by the MOCVD system (hereafter InSb-MO) commenced with a 25 nm Zn-doped InSb seed layer deposited at 400 °C over 30 min, after which a 1 μm InSb layer was grown at 500 °C. The thickness of the main InSb layer was maintained at 1 μm for both growth methods. Figure 1a and b schematically depicts the layer structures for GaAs(001)/InSb­(100 nm)/InSb­(1 μm) grown by MBE and GaAs(001)/Zn:InSb­(25 nm)/InSb­(1 μm) grown by MOCVD, respectively.

**Figure 1:**
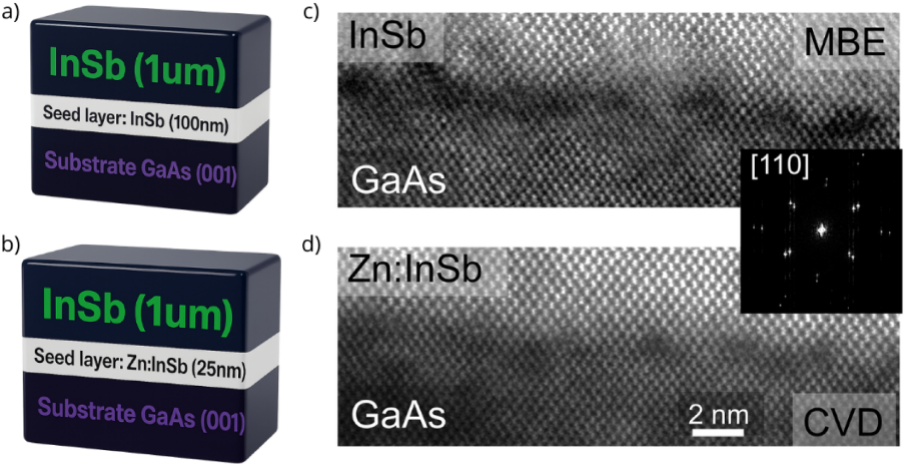
Schematic illustration of the InSb epilayer grown on GaAs by (a) MBE and (b) MOCVD. HR STEM images of the GaAs/InSb interface reveal the distinct growth characteristics achieved through (c) MBE and (d) CVD. The inset shows FFT analysis of the GaAs/InSb interface. Dark regions observed in both interfaces indicate the presence of various structural defects.

Raman spectroscopy was performed by using a LabRAM HR Evolution confocal Raman microscope equipped with a Peltier-cooled charge-coupled device (CCD) detector. A 532 nm semiconductor diode laser (25 mW) was used as the excitation source with a spot size of approximately 0.5 μm and a power density of 12.7 MW^–2^. The scattered light was dispersed by a 600 lines/mm diffraction grating, and a 50× long-working-distance (NIR-LWD, 100×/N.A. = 0.9) objective was used for focusing and collection.

EPR spectra were recorded by using a Bruker EMX Plus spectrometer operating in the X-band (9.45 GHz). Key parameters included a microwave power of 0.1 mW and a field modulation frequency of 100 kHz.

XRD analysis was performed using a PANalytical Empyrean diffractometer with CuKα radiation (λ = 1.5418 Å). Data were collected in a 2θ geometry from 20° to 80° with a tube voltage of 40 kV and a current of 45 mA. All Raman, EPR, and XRD measurements were performed at room temperature.

The samples were subjected to cumulative gamma irradiation using a Gammacell 220 Excel irradiator (^60^Co source) at a dose rate of 2.7 kGy/h. The irradiation was performed at room temperature in ambient air. The cumulative doses administered were: 0, 1, 5, 10, 15, 20, and 40 kGy. This range covers the spectrum from mild exposure (typical of short-duration satellite missions) to severe degradation (typical of multiyear Jovian missions or reactor cores).^28^

TEM lamellae were prepared using a Thermo Fisher Helios NanoLab 650 focused ion beam (FIB) instrument. To preserve the sample’s surface integrity, a 100 nm platinum (Pt) protective layer was first deposited via an electron beam (e-beam). This was followed by a 2 μm Pt layer deposited by using a gallium ion (Ga-ion) beam. A final low-energy cleaning step was performed with a 2 keV Ga-ion beam to minimize amorphization.

Imaging was conducted on a probe-corrected Thermo Fisher Titan 80-300 microscope operating at 200 keV. The images were acquired in scanning transmission electron microscopy (STEM) mode by using a high-angle annular dark-field (HAADF) detector.

## Computational Details

The theoretical analysis of the GaAs/InSb interface was conducted using Density Functional Theory (DFT)^35,36^ as implemented in the Vienna Ab initio Simulation Package (VASP).^37^ The exchange and correlation potentials were described using the generalized gradient approximation (GGA), as parametrized by Perdew, Burke, and Ernzerhof (PBE)^38^ and van der Waals (vdW) interactions were treated within the semiempirical Grimme D3 scheme (PBE-D3).^39^ The projector-augmented wave (PAW) method was employed to model the interactions between valence electrons and ionic cores.^38,40^ Brillouin Zone integrations were conducted using a 4 × 4 × 1 Γ-centered Monkhorst–Pack sampling mesh for structural optimization, while a 20 × 20 × 1 k-point grid was employed for Projected Density of States (PDOS) calculations.^41^ Spin–orbit coupling (SOC) was considered only in the PDOS calculation through a single-point run. The results show no qualitative change compared to the PBE PDOS calculations shown in Figure 2c. The SOC-resolved PDOS is provided in the Supporting Information, Section Spin–Orbit Coupling Analysis.

**Figure 2:**
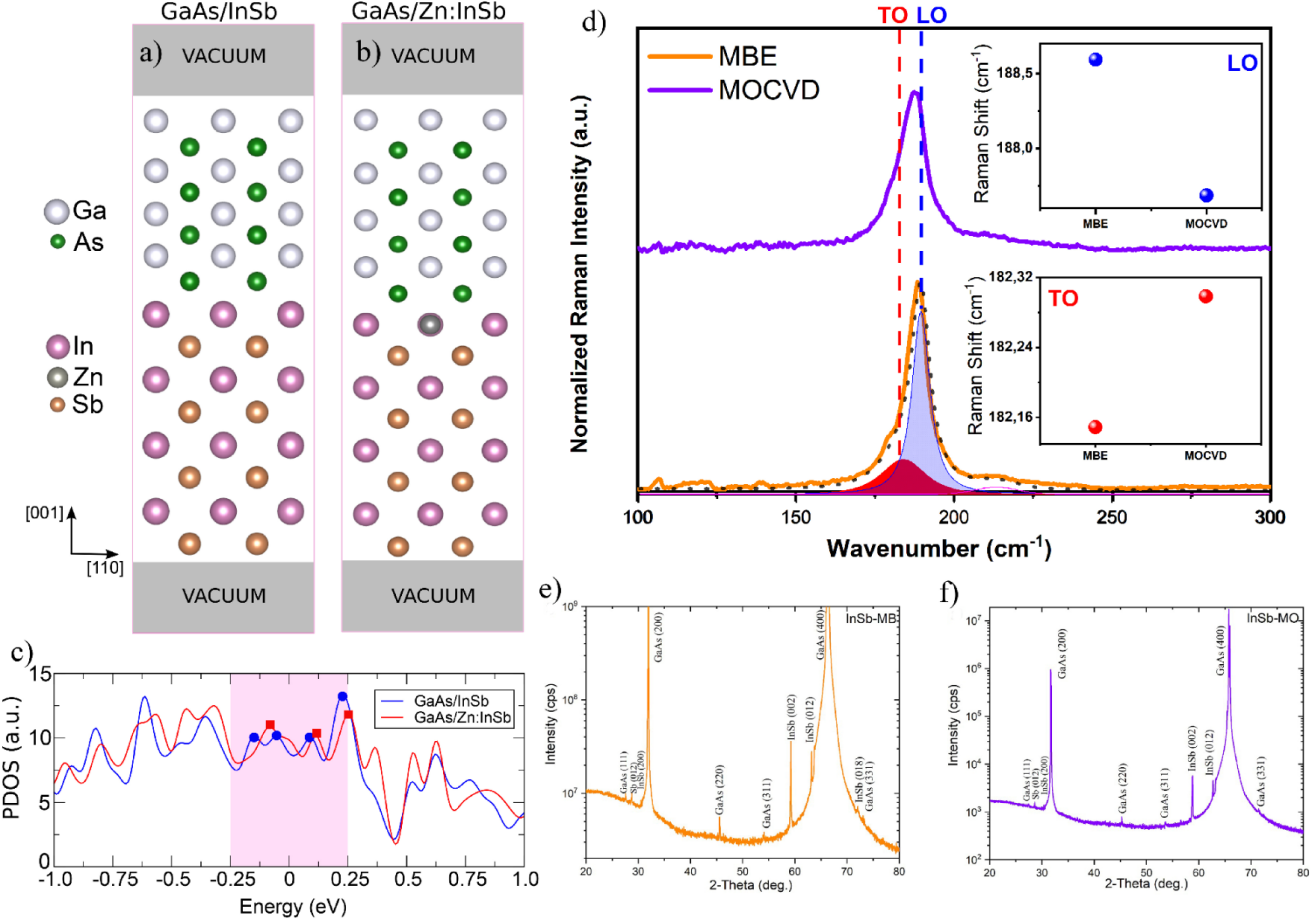
Structural models of the (a) pristine GaAs/InSb (left) and (b) GaAs/Zn:InSb (right) interfaces, where Zn atoms substitute for sites in the InSb region. Ga, As, In, Zn, and Sb atoms are represented by white, green, magenta, gray, and orange spheres, respectively. (c) Projected density of states (PDOS) for the GaAs and InSb layers closest to the interface, comparing the pristine case (blue) with the Zn-doped interface (red). The highlighted energy window (−0.25 to 0.25 eV) around the Fermi level (*E* = 0 eV) corresponds to the region where interfacial trap states are identified, marked by circles for the pristine interface and squares for the Zn-doped interface. (d) Raman spectra of InSb samples grown by MBE (orange) and MOCVD (purple). The curves under the spectra represent the Lorentzian functions used for the deconvolution of the Raman bands. Within the graph are shown the positions of the TO and LO peaks. (e) and (f) XRD patterns of InSb-MB and InSb-MO samples, respectively.

The electronic wave functions were expanded using a plane-wave basis set with an energy cutoff of 400 eV. The GaAs/InSb interface was modeled using supercells oriented in the *x*–*y* plane, featuring a 2 × 2 lateral periodicity with 128 atoms. The structure consisted of an 8-monolayer (ML) InSb slab stacked on top of an 8-ML GaAs slab, aligned along the <001> crystallographic direction. To eliminate spurious interactions between periodic images of the supercell, a 15 Å vacuum region was introduced along the same direction. During structural minimization, all atoms were allowed to relax, with the exception of the first two monolayers adjacent to the vacuum, which were held fixed.

The ∼15.45% lattice mismatch between GaAs and InSb, referenced to bulk GaAs, markedly increases the computational cost of simulating realistic GaAs/InSb interfaces, making DFT analysis computationally intensive. Given that our primary goal was to investigate how the Zn impurity in the InSb slab affects the states near the Fermi level, all strain was applied to the GaAs slab to manage this computational cost. To model GaAs/InSb–Zn, the Zn impurity was introduced at an In site at the interface in the InSb region, selected based on cohesive energy calculations. This setup resulted in a Zn concentration of 3.125% relative to that of the In sublattice. This value corresponded to 0.78125% with respect to the entire slab and 12.5% within the interface layer itself.

## Results and Discussion

High-resolution scanning transmission electron microscopy (HR STEM) was used to analyze the cross-section of the GaAs/InSb interface (Figure 1c,d). As expected from the ∼15% lattice mismatch, the images reveal various defects concentrated near the interface, consistent with previous reports.^42–47^ The inset, a fast Fourier transform (FFT) of the interface, confirms this mismatch by showing two distinct reflections for each Bragg diffraction.

The incorporation of Zn at the InSb interface enhances interfacial quality, evidenced by the suppression of defects (dark-contrast regions) in the MOCVD-grown sample, a finding further corroborated by DFT analysis. Previous studies investigating InSb doping report a maximum lattice mismatch reduction of approximately 0.05%.^48^ Given that the estimated resolution of the FFT analysis is ∼10%, quantifying such minute strain variations is unfeasible. Consequently, Zn incorporation does not appear to induce a substantial reduction in the lattice mismatch. Therefore, the observed structural improvement is primarily attributed to defect accommodation and interfacial chemical reconfiguration, whereas the contribution of lattice mismatch reduction is likely negligible.

To elucidate the microscopic mechanisms underlying the disparities between InSb-MO and InSb-MB samples, Density Functional Theory (DFT) calculations were performed, focusing specifically on the role of Zn impurities in modifying the interfacial properties. Figure 2a depicts the atomic interface models for the GaAs/InSb (MBE sample) and GaAs/Zn:InSb (MOCVD sample) heterostructures, with computational details provided in Supporting Information, Section I­(A).

To elucidate the nature of interfacial bonding, the electronic structure was characterized via differential charge density, Bader charge analysis, and projected density of states (PDOS). Figure 2c displays the PDOS spectra of the GaAs and InSb layers adjacent to the interface, facilitating a direct comparison between the pristine and Zn-doped GaAs/InSb systems. While both systems exhibit a similar overall electronic structure, distinct deviations emerge in the vicinity of the Fermi level. In the pristine case, four trap states (blue circles) are identified within the −0.25 to 0.25 eV range, whereas the Zn-doped interface exhibits only three (red squares), corresponding to an ∼25% reduction in the density of gap states. These trap states appear as peaks in the PDOS. Although the numerical decrease may appear modest, it is highly relevant because these localized states are located precisely at energies that dominate charge transport and recombination processes.

Beyond the trap count, the PDOS intensity within this energy range is also reduced for the Zn-doped interface, indicating partial passivation of the remaining localized states. To understand the origin of this passivation, we analyzed the underlying mechanism. Substitutional Zn in the InSb sublattice behaves as an acceptor, which lowers the Fermi level relative to that of GaAs. This causes electrons to flow from the Zn:InSb side to the GaAs side of the interface. Our Bader charge analysis confirms this mechanism quantitatively (see Supporting Information, Section II), the total charge transfer (CT) across the interface increases from 1.50 × 10^14^ e/cm^2^ in the pristine system to 1.54 × 10^14^ e/cm^2^ upon Zn doping. This flow of electrons neutralizes the interfacial states, thereby reducing the density of traps observed in the PDOS.

Collectively, the PDOS data in Figure 2c, substantiated by differential charge density and Bader charge analyses (Supporting Information, Section II), demonstrate that Zn doping optimizes the interfacial electronic landscape by mitigating trap state density and enhancing interfacial charge transfer. This synergistic mechanism mitigates carrier localization, suppresses scattering and recombination pathways, and is consequently expected to facilitate efficient charge transport across the GaAs/InSb heterointerface. This improvement indicates that the InSb-MO sample exhibits higher interfacial quality compared to the InSb-MB sample.

To investigate the difference between the InSb-MO and InSb-MB samples, Raman spectra were collected in the range of 100–300 cm^–1^ using a 532 nm excitation laser at 25 mW power. The vibrational spectrum of bulk InSb is well established, typically exhibiting a Transverse Optical (TO) phonon mode at ≈180 cm^–1^ and a Longitudinal Optical (LO) phonon mode at ∼191 cm^–1^.^49–55^ In the measured spectra, the LO phonon modes were observed at 188.6 cm^–1^ (InSb-MB) and 187.6 cm^–1^ (InSb-MO), while the TO modes appear at 182.1 cm^–1^ (InSb-MB) and 182.3 cm^–1^ (InSb-MO), respectively, as displayed in Figure 2d.

An additional noteworthy difference between the samples lies in the full width at half-maximum (FWHM) of the TO phonon mode: the InSb-MO sample exhibits a narrower peak (10.14) compared to the InSb-MB sample (16.73), a change of around 61%. Narrow Raman linewidths correspond to well-defined vibrational modes, indicative of high crystalline quality and long-range order. Conversely, spectral broadening signals the presence of structural disorder, defects, or impurities. This observation aligns with the DFT analysis and the *g*-factor values derived from EPR experiments (discussed below), collectively corroborating the superior crystalline quality of the InSb-MO sample.

The enhanced crystalline quality of the InSb-MO sample is attributed to the distinct growth methodologies employed. Specifically, the MOCVD architecture incorporates a Zn-doped InSb seed layer, a feature absent in its MBE counterpart. This seed layer reduces the lattice mismatch between InSb and the GaAs substrate, thereby decreasing the density of dislocations at the interface, which likely contributes to the improved structural and electronic properties observed in the MOCVD-grown film.

The X-ray diffraction (XRD) pattern of the InSb-MB sample exhibits a series of well-defined peaks (Figure 2e), indexed to specific crystallographic planes. Reflections observed at 27.57° (111), 31.90° (200), 45.50° (220), 54.13° (311), 66.36° (400), and 73.10° (331) confirm the single-crystalline nature of the GaAs substrate.^56,57^ Diffraction peaks associated with the InSb epilayer are identified at 30.53° (200), 59.34° (002), 64.00° (012), and 72.10° (018). The observation of multiple InSb reflections suggests that the film is not purely epitaxial with a single orientation but rather polycrystalline or composed of domains with varied orientations. Notably, a prominent secondary peak detected at 28.77° corresponds to the elemental antimony (Sb) phase with (012) orientation. The existence of this phase points to nonstoichiometric growth conditions,^58^ resulting in an excess of unreacted Sb. Similarly, the diffractogram of the InSb-MO sample reveals contributions from both the GaAs substrate and the InSb layer (Figure 2f). Substrate reflections were located at 27.44° (111), 31.71° (200), 45.43° (220), 54.60° (311), 65.80° (400), and 71.60° (331). InSb-related peaks were resolved at 30.37° (200), 58.80° (002), and 62.75° (012). The secondary Sb (012) peak at 28.62° is also present, indicating nonstoichiometry similar to that of the MBE sample. However, in contrast to the InSb-MB spectrum, the InSb (018) reflection at ≈72° is absent in the InSb-MO sample. The presence of this specific reflection in the InSb-MB sample implies a higher degree of polycrystallinity or a more complex domain architecture, potentially indicating an inferior structural uniformity. This discrepancy is likely attributable to distinct strain relaxation mechanisms operative during the growth of the thicker layer (100 nm).^59^

The presence of the Sb (012) peak in both samples indicates that, irrespective of the growth technique (MBE or MOCVD), an excess of antimony exists. The formation of this secondary phase significantly impacts electronic properties and defect density, corroborating the EPR and Raman results, where pronounced *g*-factor anisotropy was observed.^60^ Conversely, the absence of the InSb (018) peak in the MOCVD sample suggests that the 25 nm Zn:InSb seed layer promoted enhanced crystallinity. This superior structural quality aligns with those of our HR-STEM and DFT analyses.

Figure 3a,b shows the EPR signals of InSb-MB and InSb-MO samples as a function of static magnetic field, respectively, with the magnetic field applied perpendicular to the film plane in both cases. The linewidth (Δ*H*) values obtained from the first-derivative EPR spectra are 3.72 kOe for InSb-MB and 3.12 kOe for InSb-MO. The peak-to-peak amplitude (Δ*A*) is also markedly different between the two samples: the InSb-MO film exhibits a more intense signal (Δ*A* = 11.91) compared with the MBE-grown film (Δ*A* = 7.89). These findings indicate that the MOCVD-grown sample displays a narrower and stronger EPR response, suggesting that it possesses superior structural quality than the MBE growth one. This interpretation is consistent with the well-established relationship between EPR linewidth and crystalline disorder, narrow linewidths are typically associated with reduced defect densities and weaker inhomogeneous broadening mechanisms. For example, conduction-electron ESR in high-quality bulk n-type InSb shows linewidths as small as 0.3 G at X-band frequencies,^13^ while even moderately doped or stressed crystals tend to retain linewidths in the few-gauss range.^61^ In contrast, the much broader linewidths observed in our thin-film samples (on the order of kiloOersted) can reflect the substantial presence of point defects, local strain fields, and structural inhomogeneities. Similar defect-mediated broadening has been reported in studies of spin–orbit-coupled III–V semiconductors,^62^ and is expected for epitaxial layers grown under different kinetic regimes. Furthermore, our observation that the MOCVD sample produces both a narrower Δ*H* and a higher Δ*A* is consistent with earlier reports showing that improved crystalline uniformity enhances spin coherence and increases EPR signal intensity.^63,64^ The stronger response of the InSb-MO film suggests longer spin relaxation times and fewer paramagnetic centers acting as scattering sites. This difference may arise from the distinct growth mechanisms of MOCVD and MBE, while MBE typically yields atomically abrupt interfaces and is also more sensitive to low-level contamination and surface reconstruction instabilities, whereas MOCVD can produce smoother and more homogeneous extended layers under optimized precursor flux and temperature conditions.

**Figure 3:**
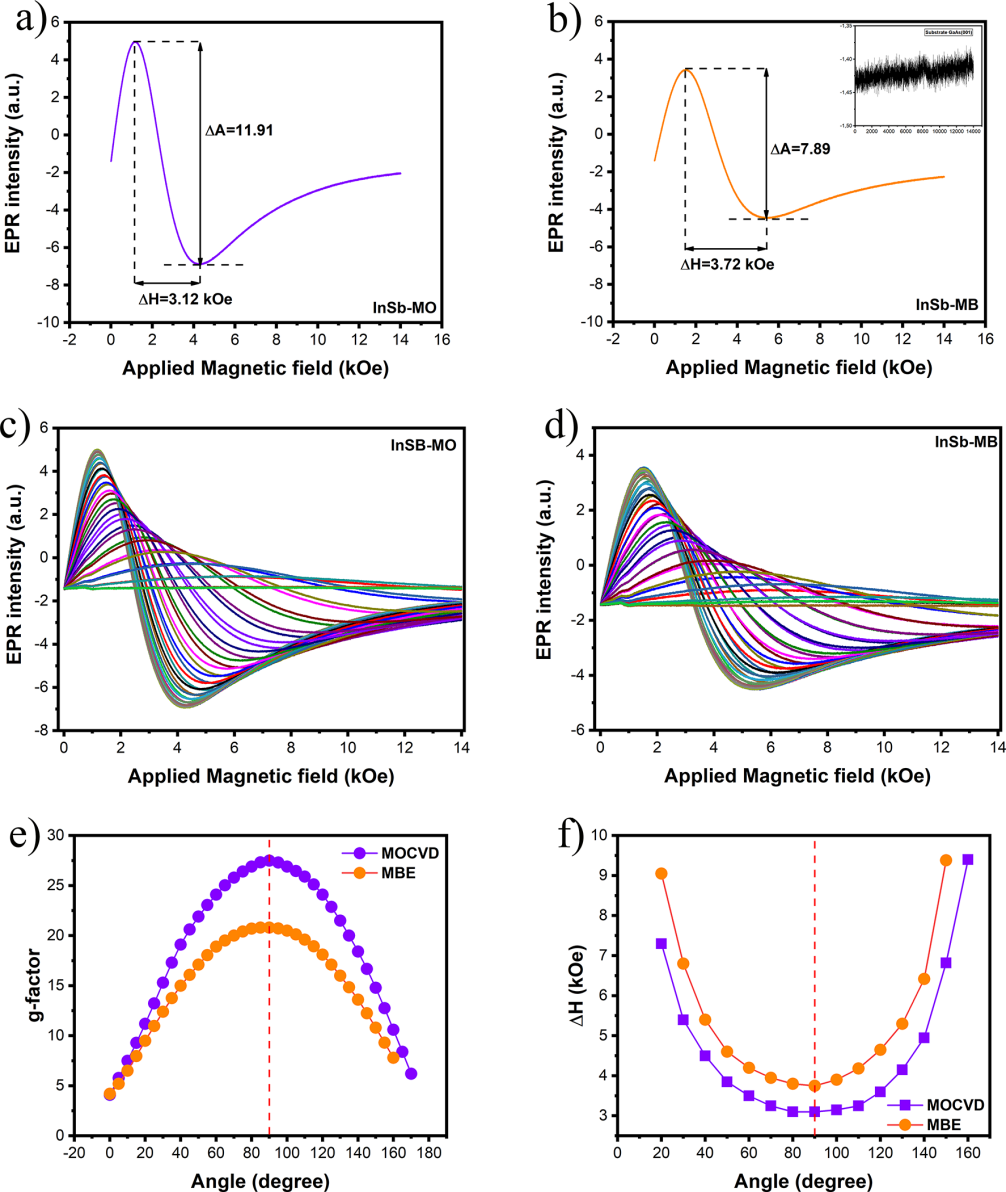
EPR signals of (a) InSb-MB and (b) InSb-MO samples as a function of the static magnetic field, respectively. Angle dependence of EPR spectra of InSb grown by (c) MBE and (d) MOCVD as a function of the static magnetic field, respectively. (e) *g*-factor and (f) Δ*H* as a function of the angle between the applied field and the film plane.

To ensure that the observed resonance originates from the InSb layers rather than from the GaAs (001) substrates, control measurements were performed on the bare substrates. As displayed in the inset of Figure 3a, the GaAs substrate exhibits no detectable resonance within the investigated magnetic-field range, confirming that the EPR signals arise exclusively from the InSb epilayers. Regarding the spectral line shape, the InSb-MO film displays a narrower linewidth compared to the MBE sample. This feature indicates superior crystalline quality, as the EPR linewidth is directly correlated with structural integrity and local disorder. The *g*-factor is a dimensionless parameter that relates the electron’s magnetic moment to the magnetic field and is defined by the resonance condition:

1 $$g=\frac{h\nu }{\beta H_{res}}$$

where *h* is the Planck constant (4.135 × 10^–15^ eV·s), ν is the frequency (9.45 GHz), β is the Bohr magneton (5.788 × 10^–5^ eV/T), and *H*_res_ is the applied magnetic field at resonance. At θ = 90°, where the applied field is perpendicular to the film plane, the measured *g*-factor values are *g* ≈ 20.8 for InSb-MB and *g* ≈ 27.5 for InSb-MO, in agreement with the fitted *g*_⊥_ components. These values are smaller in magnitude than the bulk Landé factor |*g*| ≈ 51 for high-purity InSb, as determined from cyclotron resonance and time-resolved spin-precession measurements,^65–68^ but they fall within the range reported for heavily doped epilayers and quantum-confined InSb structures, where band nonparabolicity and high electron concentrations renormalize g* toward lower absolute values.^69,70^

Notably, the InSb-MO sample exhibits a larger *g*-factor compared to InSb-MB. This difference may arise from variations in electron density. Theoretical models predict an inverse relationship between the effective *g*-factor and electron density due to band nonparabolicity. In our heterostructures, the carrier (electron) density values were determined as 1.82 × 10^12^ cm^–3^ and 1.19 × 10^12^ cm^–3^ for InSb-MB and InSb-MO, respectively. Consequently, the lower *g*-factor observed in the InSb-MB sample corresponds to its higher electron density, a finding that is in good agreement with theory.^13^ The reduction in |g*| with increasing carrier density and wave vector *k* is a well-established consequence of the strong conduction-band nonparabolicity in narrow-gap III–V semiconductors, which enhances conduction-valence band mixing and modifies the Zeeman splitting.^68,70^

Figure 3c,d shows the angular dependence of the EPR spectra recorded by rotating the sample from 0° to 180° in 5° steps for InSb-MB and InSb-MO, respectively. The angle θ is defined as the angle between the film plane and the external magnetic field. The *g*-factor values were extracted for all angles and are plotted in Figure 3e for both samples. Both films exhibit distinct angular anisotropy. As expected, the *g*-factor values reach a maximum at 90°. The angular dependence of the effective *g*-factor is well described by an axially symmetric tensor of the form:

2 $$g_{res}=\sqrt{g_{⊥}^{2}\sin^{2}\theta +g_{∥}^{2}\cos^{2}\theta }$$

where *g*_⊥_ and *g*_∥_ denote the principal components of the tensor with the field perpendicular and parallel to the film plane, respectively. By fitting the experimental data using eqs 1, 2 and a nonlinear least-squares fit of *g*(θ) for both samples, we extract *g*_⊥_, *g*_∥_. Anisotropy is typically quantified by the difference between the principal components.

3 $$\Delta g=g_{⊥}-g_{∥}$$

In solid-state systems, the *g*-factor anisotropy serves as a powerful spectroscopic probe of the local symmetry and structural defects at the site of the paramagnetic center. It arises primarily from the spin–orbit coupling (SOC) of the unpaired electron, which is significant in semiconductors containing heavy elements like indium and antimony (InSb). When the local environment of the spin deviates from cubic symmetry, the *g*-tensor becomes anisotropic, with the magnitude of the anisotropy, Δ*g*, providing a quantitative measure of this distortion.^71^ The fitted values together with their 95% confidence intervals, as well as the coefficients of determination (*R* ^2^) and root-mean-square (RMS) residuals of the fits, are summarized in Table S2 (Supporting Information). For both InSb-MB and InSb-MO, *R* ^2^ values are very close to unity and the residuals show no systematic angular trend, confirming that the standard axially symmetric *g*-tensor provides an adequate description of the experimental angular dependences.

The magnitude of the *g*-factor anisotropy observed in our study, Δ*g* ≈ 0.67 for InSb-MB and Δ*g* ≈ 0.19 for InSb-MO, is fully consistent with the presence of strong spin–orbit coupling (SOC) in InSb and with the symmetry breaking imposed by epitaxial strain and interfacial disorder.^72,73^ In InSb quantum wells and nanowire quantum dots, comparable or even larger anisotropies between in-plane and out-of-plane *g*-components (|*g*_⊥_| ∼ 20–30 vs |*g*_∥_| ∼ 50) have been reported and attributed to SOC combined with structural inversion asymmetry and interface roughness.^74–76^ Our results extend these observations to lattice-mismatched InSb epilayers on GaAs: the larger Δ*g* in the MBE-grown film indicates a more distorted and strained local environment at the paramagnetic centers, consistent with the higher density of threading dislocations and Sb-rich secondary phases identified by XRD, Raman, and HR-STEM. In contrast, the smaller Δ*g* of the MOCVD-grown film reflects a more isotropic electronic environment, which we attribute to the improved interfacial quality produced by the Zn:InSb seed layer. Therefore, the EPR data provide robust evidence that a sharper and more coherent interface leads to a more isotropic electronic environment for the spin centers in the InSb film.

The EPR linewidth Δ*H*(θ) further clarifies the role of inhomogeneous broadening, as shown in Figure 3f, where the EPR linewidth (Δ*H*) was analyzed as a function of measurement angle for both samples. Whereas high-quality bulk *n*-InSb can exhibit conduction-electron ESR linewidths of the order of a few gauss at X-band frequencies,^77^ the linewidths observed in our epilayers are in the kilo-oersted range, which is indicative of substantial local disorder and distributions of *g*-values. Such broad lines are typical when the *g*-tensor is spatially inhomogeneous due to a distribution of local strain fields, composition fluctuations, and defect complexes, and are commonly interpreted as inhomogeneous broadening in solid-state EPR.^78,79^ The systematically broader Δ*H* in the InSb-MB sample, together with its larger Δ*g*, points to a wider distribution of local environments around the spin centers, while the narrower and less anisotropic linewidth in InSb-MO is consistent with the higher structural uniformity inferred from our STEM, XRD, and Raman analyses.

Subsequently, the evolution of the magnetic properties under gamma irradiation was monitored via EPR measurements. Figure 4a,b displays the EPR spectra for both samples subjected to doses ranging from 0 to 40 kGy, with the angle between the film plane and the applied magnetic field fixed at 90°. As detailed in Section III of the Supporting Information, for the InSb-MO sample, the linewidth decreases by approximately 7% when comparing the pristine state (0 kGy) to the 1 kGy dose. Upon increasing the dose to 5 kGy, the signal intensity increases markedly, and the linewidth broadens to 3.4 kOe, representing an increase of about 18%. Notably, at 10 kGy, both the EPR signal intensity and linewidth increase further (reaching 4.3 kOe), corresponding to an ∼20% variation. However, at higher cumulative doses (20 and 40 kGy), the EPR signal weakens and the linewidth decreases to 3.3 and 2.6 kOe, respectively. Furthermore, the InSb-MO sample exhibited an initial significant rise in amplitude (Δ*A*), from 11.91 at 0 kGy to 20 at 1 kGy, 26 at 5 kGy, and 36 at 10 kGy. This increase is consistent with the generation of paramagnetic centers due to radiation-induced defects such as vacancies and interstitials. However, as the dose was further increased to 20 and 40 kGy, the amplitude showed a notable decline to 15 and 5 au, respectively. This decrease suggests that at higher doses, the radiation may induce defect aggregation or radiation-induced annealing, thereby transforming paramagnetic centers into nonparamagnetic ones.^80,81^

**Figure 4:**
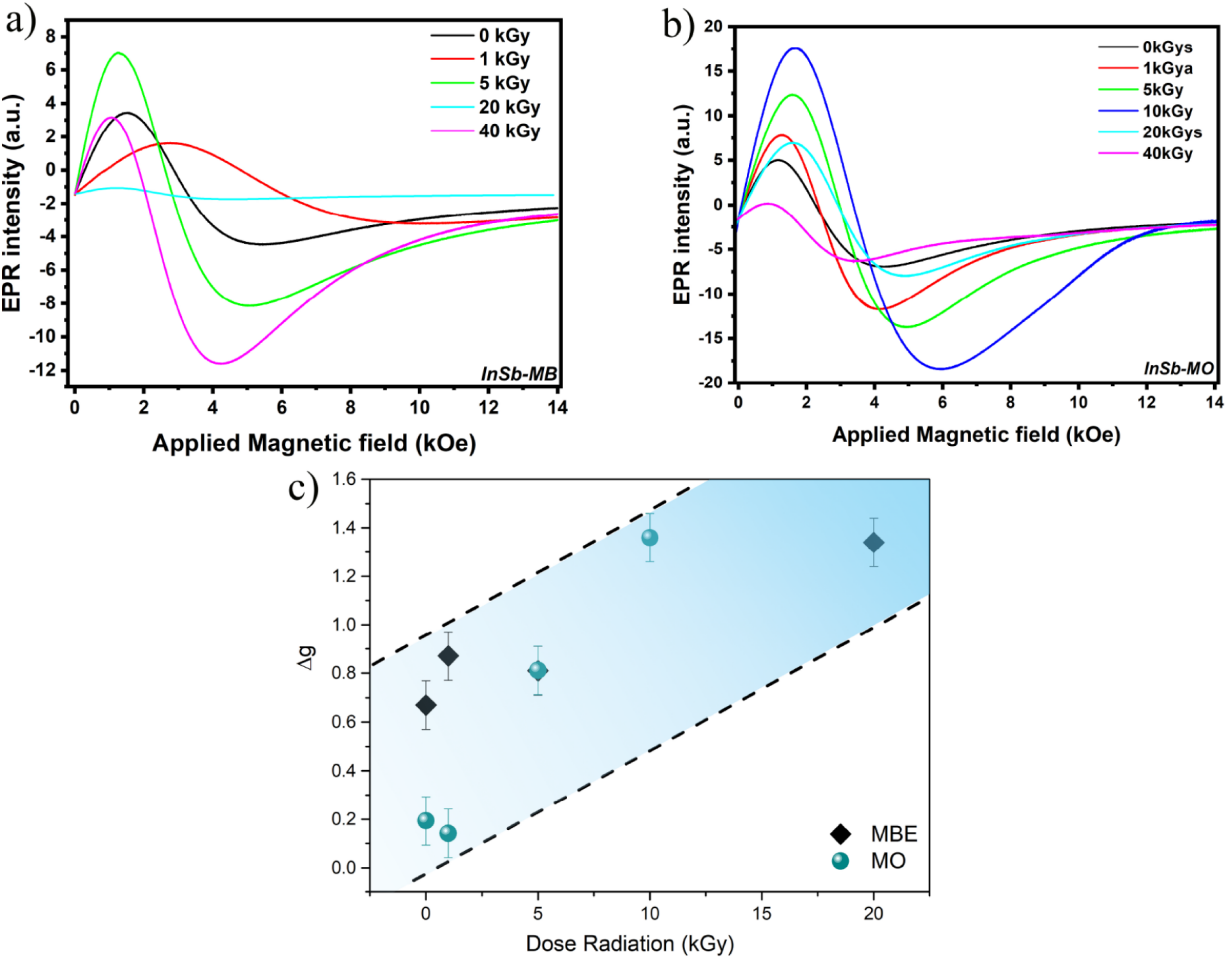
EPR signals of (a) InSb-MB and (b) InSb-MO samples under various γ radiation doses, respectively. (c) The corresponding Δ*g* as a function of irradiation.

Conversely, the InSb-MB sample exhibits pronounced changes in response to gamma irradiation. At 1 kGy, the EPR signal intensity fluctuates and the linewidth increases significantly to 7.1 kOe, representing a more than 2-fold broadening relative to the unirradiated sample. At 5 kGy, the signal increases and the linewidth decreases by almost 50%. Notably, at 20 kGy, the signal is nearly quenched, yet at 40 kGy it reemerges with a linewidth of approximately 3.2 kOe. Its amplitude initially decreased from 7.89 at 0 kGy to 5 at 1 kGy and increased to 15.5 at 5 kGy, indicating a rise in spin concentration. While the amplitude dropped to 0.67 at 20 kGy, a substantial increase to 15 was observed at the highest dose of 40 kGy. This behavior suggests a more complex dose-dependent mechanism of defect formation, where a different type of paramagnetic defect might become dominant or more stable at very high radiation doses. The stark difference in the amplitude behavior between the two samples highlights that their distinct initial microstructural landscapes lead to different responses to radiation-induced damage.^80,82^

The observed nonlinear dependence of EPR signal intensity on radiation dose can be attributed to the complex interaction between gamma photons and lattice defects. As the EPR signal arises from unpaired spins, its integrated intensity is directly proportional to the concentration of paramagnetic centers including free charge carriers. γ Radiation induces ionization events by transferring sufficient energy to rupture bonds within the crystal lattice. Consequently, electron–hole pairs are generated via excitation from the valence to the conduction band. However, in the presence of structural defects (such as vacancies, interstitials, dislocations, or impurities), these carriers are prone to trapping, thereby altering the effective spin population.

Upon carrier localization at defect sites, electrons are withdrawn from the itinerant carrier population and may subsequently recombine with holes, resulting in the formation of diamagnetic states and a consequent decrease in the EPR signal intensity. This carrier trapping mechanism effectively accounts for the observed nonmonotonic behavior: at lower doses, radiation-induced ionization enhances the free carrier concentration; at intermediate doses (10–20 kGy), carrier compensation via trapping dominates, leading to signal suppression; whereas at higher doses (40 kGy), the generation of new paramagnetic centers or the activation of deep-level defects may restore the EPR response. The near-complete quenching of the InSb-MB EPR signal at 20 kGy suggests that carrier compensation mechanisms prevail at this fluence, highlighting the critical role of radiation-induced defects in governing the magnetic properties of these heterostructures.

In semiconductor systems, the *g*-factor anisotropy serves as a sensitive probe of atomic-level defects and lattice distortions. γ Radiation generates point defects—such as vacancies and interstitials—that break local symmetry and modify the electronic environment of the spin centers. As shown in Figure 4c, irradiation induced a significant increase in the *g*-factor anisotropy in both samples. The Δ*g* value for the InSb-MO sample reached 1.36 after 10 kGy of irradiation, while that of the MBE-grown sample reached 1.34 after 20 kGy. These results indicate a more distorted and asymmetric environment for the spins in the irradiated samples, which aligns directly with a reduction in crystal lattice quality due to radiation damage. Mechanistically, γ radiation transfers momentum to lattice atoms via high-energy scattering events, displacing them from their equilibrium positions. This process leads to the generation of Frenkel pairs, consisting of a vacancy and an interstitial atom^83^ In systems containing heavy elements like InSb, the strong spin–orbit coupling makes the *g*-tensor particularly sensitive to such structural perturbations. The unpaired electrons can then localize at defect sites, such as vacancies, antisites (e.g., In on Sb sites), or interstitial atoms, which naturally lack the cubic symmetry of the original lattice. The local symmetry breaking of these new paramagnetic centers is directly reflected in the observed increase in *g*-factor anisotropy.^84^

However, the EPR results reveal that the Zn-doped InSb (InSb-MO) films exhibit a markedly higher sensitivity to gamma irradiation compared to their undoped InSb-MB counterparts. This enhanced susceptibility is attributed to the incorporation of Zn atoms within the crystal lattice. Given that Zn possesses a substantially lower atomic mass than In or Sb, it is more susceptible to displacement via momentum transfer from gamma-induced secondary electrons, thereby increasing the probability of Frenkel pair generation. Moreover, since Zn acts as a substitutional dopant with distinct bonding characteristics, its displacement induces greater local lattice instability, facilitating the formation of complex paramagnetic centers. Furthermore, Zn introduces shallow acceptor levels that may serve as precursors for radiation-induced trapping or recombination centers, thereby amplifying the spectral modifications observed in EPR. Collectively, these factors elucidate why the Zn-containing films display more pronounced dose-dependent variations in resonance field, linewidth, and integrated signal intensity relative to the undoped samples.

The effect of gamma-ray irradiation on the crystal lattice was further probed via Raman scattering spectroscopy. The normalized Raman spectra for the InSb-MB and InSb-MO samples, acquired after exposure to gamma-ray doses of 0, 1, 5, 10, 20, and 40 kGy, are presented in Figure 5a,b. In the pristine state, both films exhibit the characteristic longitudinal optical (LO) and transverse optical (TO) phonon modes of InSb centered at approximately 189 cm^–1^ and 182 cm^–1^, respectively, attesting to their high crystalline quality.

**Figure 5:**
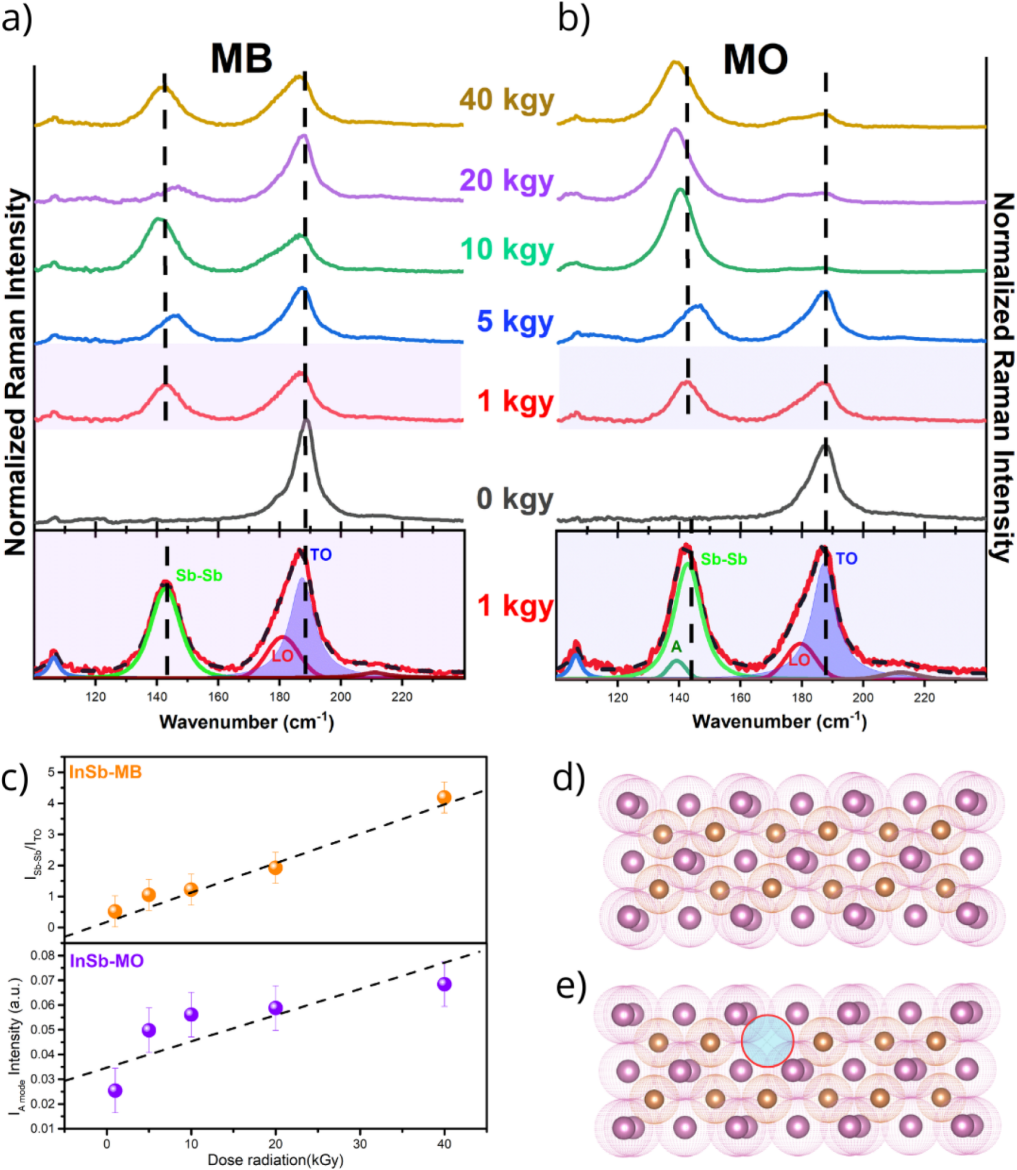
(a) Normalized Raman spectra of the InSb-MBE and (b) InSb-MOCVD samples as a function of increasing γ radiation dose (1, 5, 10, 20, and 40 kGy). The deconvolution using Lorentzian functions for the 1 kGy dose is shown below the respective spectra to illustrate the emergence of new phonon modes. (c) The intensity ratio between *I*_Sb–Sb_/*I*_TO_ (InSb-MB sample) and the intensity of the amorphous mode A (InSb-MO sample) as a function of radiation dose. (d) Before radiation and (e) after radiation, schematically illustrating the displacement of atoms and the subsequent formation of defects and homopolar Sb–Sb bonds.

Following gamma irradiation, new Raman scattering features emerge, providing direct evidence of radiation-induced structural damage. These atomic-level modifications are schematically depicted in the lattice diagrams of Figure 5d (pristine) and Figure 5e (irradiated), illustrating atomic displacement and defect formation. Notably, an additional Raman mode appears at ∼145 cm^–1^ in both samples. This peak is widely attributed to the formation of homopolar Sb–Sb bonds,^49,50^ a direct consequence of lattice disruption in InSb. Mechanistically, high-energy photons displace indium atoms from their equilibrium sites, generating Frenkel pairs (In vacancies and interstitials) and leaving a local excess of antimony atoms that subsequently relax into Sb–Sb bond configurations. The progressive enhancement of this mode correlates directly with the radiation dose, reflecting the accumulation of Sb–Sb defects. This trend is quantified in Figure 5c (top panel), which plots the integrated intensity ratio of the Sb–Sb band to the reference TO mode, highlighting a more pronounced defect generation in the InSb-MB sample.

Additionally, a distinct shoulder peak, denoted as Mode A, was detected at ∼139 cm^–1^ in the InSb-MO samples, exhibiting a linear correlation between the intensity and radiation dose (see Figure 5c, bottom panel). This feature is characteristic of disorder-activated scattering and has been widely reported in amorphous semiconductors such as Si, Ge, and GaAs.^51^ Its presence signals localized amorphization or high structural disorder, which relaxes the *q* = 0 selection rule and activates the longitudinal acoustic (LA) phonon branch, likely driven by cumulative stress from radiation-induced defects. The significant increase in the intensity of the ∼140–145 cm^–1^ complex in MOCVD samples at high doses further underscores the extensive lattice damage, marking a clear transition from a crystalline to a highly disordered state. Concurrently, a striking feature is the near-total suppression of the characteristic TO and LO bands as the radiation dose increases.

The introduction of defects and localized strain—intrinsic consequences of gamma irradiation—compromises the long-range order of the crystal lattice. This structural perturbation modifies the effective interatomic force constants, thereby altering the vibrational modes. Consequently, the characteristic phonon bands of InSb exhibit both mode softening (redshift) and spectral broadening. This effect is evident in the Raman spectra of both InSb-MOCVD and InSb-MBE samples (see Supporting Information, Figure S5), which display a systematic redshift and asymmetric broadening of the Transverse Optical (TO) mode with increasing gamma dose. Such phonon softening serves as a quantitative proxy for lattice damage and aligns with established phenomena in irradiated III–V semiconductors.^49,50^

The combined analysis from EPR and Raman spectroscopy yields a comprehensive assessment of the radiation-induced damage in the InSb samples, revealing a correlation between the applied gamma dose and the degradation of the crystal lattice.

High-energy γ radiation interacts with the crystal lattice primarily through Compton scattering, the photoelectric effect, and pair production, processes that collectively generate energetic secondary electrons within the material.^85^ These electrons, in turn, transfer kinetic energy to the lattice atoms via elastic collisions, displacing them from their equilibrium positions to generate Frenkel pairs, consisting of a vacancy and a corresponding interstitial atom.^86,87^ The energy required to displace an atom, known as the displacement threshold energy (*E*_*d*_), is a critical parameter. For typical semiconductors, the displacement energies for different sublattices typically lie in the range of tens of electron volts (eV). Considering the high energy of the incident gamma photons (1.17 and 1.32 MeV), the available energy is orders of magnitude above the displacement thresholds for both indium and antimony atoms in the InSb lattice, implying that both sublattices are susceptible to displacement damage.

For Indium (*Z* = 49) and Antimony (*Z* = 51), and at energies ∼1.2 MeV, Compton Scattering is the dominant interaction mechanism.^32^ In this process, the incident photon collides inelastically with a loosely bound orbital electron. The photon transfers a portion of its energy to the electron, which is ejected from the atom.^27^ This ejected electron is referred to as a “Compton electron” or secondary electron.

4 $$E_{e}=E_{\gamma }\left[1-\frac{1}{1+\frac{E_{\gamma }}{m_{e}c^{2}}\left(1-\cos\theta \right)}\right]$$

Where *E*_*e*_ is the energy of the scattered electron, *E*_γ_ is the incident photon energy, and θ is the scattering angle. For 1.2 MeV photons, these secondary electrons can have kinetic energies up to ∼1 MeV.

It is crucial to understand that the gamma photons themselves rarely cause direct displacement of lattice nuclei because they carry very little momentum. The damage is done almost exclusively by the high-energy secondary electrons generated via Compton scattering. These secondary electrons traverse the InSb lattice, losing energy through ionization (inelastic collisions with other electrons) and, occasionally, through elastic Coulombic collisions with atomic nuclei. If the energy transferred to a nucleus (*T*) during such a collision exceeds (*E*_*d*_), the atom is knocked off its lattice site. The maximum energy transfer (*T*_max_) from an electron of energy *E*_*e*_ to a nucleus of mass *M* is given by relativistic kinematics:

5 $$T_{\max}=\frac{2E_{e}\left(E_{e}+2m_{e}c^{2}\right)}{Mc^{2}}$$

For InSb:Indium (*M* ≈ 114.8 u), Antimony (*M* ≈ 121.7 u) using *E*_*e*_ ≈ 1 MeV, the maximum energy transferable to an In or Sb nucleus is on the order of tens to hundreds of eV. The displacement threshold energy (*E*_*d*_) for III–V semiconductors is typically in the range of 6–10 eV. Since *T*_max_ ≫ *E*_*d*_, a single secondary electron can displace an atom with high probability.^88^ Furthermore, if the displaced atom (Primary Knock-on Atom, PKA) receives sufficient energy, it can subsequently displace its neighbors, triggering a collision cascade, although this effect is less pronounced with electron irradiation compared to heavy ions. The result of this process is the formation of Frenkel Pairs: a vacancy left behind (*V*_In_ or *V*_Sb_) and an interstitial atom lodged in a nonlattice site (*I*_In_ or *I*_Sb_).

However, the probability of atomic displacement (vacancy and interstitial) is nonuniform. The effective displacement cross-section is a function of the transferred energy and is governed by the mass of the target atom. Although the atomic cross-section of Sb is approximately 6% larger than that of In, the displacement probability and nature of the resulting defects are complex, depending on the specific energy transfer dynamics within the lattice. Our findings indicate that γ radiation induces significant crystallographic modifications in InSb, driving atomic displacement and generating a locally nonstoichiometric defect landscape, as schematically depicted in Figure 5e. This interpretation is corroborated by the Raman measurements, which reveal the emergence of a new mode at ∼145 cm^–1^.^49,50,89^ This mode is a clear signature of homopolar Sb–Sb bonds, a configuration that forms when a significant concentration of indium atoms is displaced, leaving an excess of antimony to reconstruct into these bonds. These radiation-induced defects, including vacancies and antisites, act as paramagnetic centers and underlie the observed increase in the EPR signal intensity and *g*-factor anisotropy.

In addition to atomistic displacement, narrow-bandgap semiconductors like InSb are susceptible to ionization-induced defect formation mechanisms, such as the Varley mechanism.^90^ This process involves multiple ionizations of an anion (Sb) via the Auger effect. If an Sb atom loses multiple electrons, it may become positively charged. Surrounded by positively charged In cations, the resulting Coulombic repulsion can violently eject the Sb atom into an interstitial position, even if the kinetic momentum transfer is insufficient for displacement. While direct displacement is the primary driver at MeV energies, the Varley mechanism and recombination-enhanced diffusion can significantly alter the final defect landscape, particularly by promoting the migration and clustering of defects even at room temperature.

Furthermore, a subsidiary but non-negligible effect of gamma irradiation is localized thermal energy deposition. This thermal effect enhances atomic mobility, potentially promoting defect diffusion, aggregation, or the activation of dopants.^91^ Although the primary mechanism for defect generation is direct atomic displacement, these thermal effects likely contribute to the observed near-suppression of the LO and TO bands and the increasing prominence of the band attributed to the amorphous mode in InSb-MO. This finding aligns with the EPR observations, complementing the spectroscopic evidence of radiation-induced structural modification. These findings are crucial for developing radiation-hardened devices based on InSb, a material vital for applications in harsh environments such as space, nuclear power plants, and high-energy physics. The methodology presented here serves as a robust framework for evaluating the radiation tolerance of InSb-based electronics, providing a scientific basis for predicting the device longevity and reliability. By correlating the spectroscopic signatures of atomic-level defects with the radiation dose, this work not only quantifies the damage but also offers a pathway to engineer materials with enhanced stability for critical applications.

Finally, a consolidated comparison of our results with recent studies on radiation effects in InSb and related semiconductor systems is presented in Table S2. This comparison highlights that, while previous works have mainly focused on device-level electrical stability under neutron, our multimodal approach uniquely correlates growth method, interfacial chemistry, and spectroscopic defect signatures, revealing a trade-off between initial crystalline quality and long-term radiation tolerance in InSb epilayers

## Conclusions

The systematic investigation presented here successfully fulfills its objective of establishing a mechanistic comparison between MOCVD- and MBE-grown InSb epilayers under gamma irradiation. By integrating EPR, Raman, STEM, and DFT, the study has not only quantified the damage but revealed the underlying atomic drivers of the material’s response. Our results highlight an “Initial Quality dichotomy”: the MOCVD growth utilizing a Zn:InSb seed layer produces a superior starting material, quantified by a narrower EPR linewidth (3.12 kOe) and negligible anisotropy (Δ*g* = 0.19). This superior crystallinity is attributed to Zn-mediated charge transfer, which effectively passivates interfacial traps; however, a divergent radiation response emerges under gamma exposure. While the MOCVD material exhibits superior initial properties, it degrades catastrophically at high doses, evidenced by signal collapse and the formation of amorphous phases. Conversely, the MBE material, which is initially more defective (Δ*g* = 0.67), exhibits a saturation and recovery behavior that suggests greater high-dose stability. The mechanism of failure is primarily driven by the displacement of Indium atoms, leading to the formation of homopolar Sb–Sb bonds, definitively identified by the emergence of the Raman mode at 145 cm^–1^. This stoichiometry violation breaks the local symmetry, driving the *g*-factor anisotropy to extreme values (Δ*g* > 1.3). Ultimately, the data suggest a critical trade-off: the very chemistry that perfects the interface at growth—Zn doping—appears to introduce a thermodynamic fragility that accelerates chemical decomposition under the nonequilibrium conditions of radiation bombardment. Consequently, for the engineering of radiation-hardened InSb devices, a “dose-budgeted” design philosophy is required, where materials are not selected solely on zero-hour performance metrics. The integration of MOCVD’s interface engineering with MBE’s bulk stability, perhaps through hybrid growth strategies, represents the next frontier in creating robust semiconductors for the harsh realities of space exploration.

## Supplementary Material



## Notes

SI The Supporting Information is available free of charge at https://pubs.acs.org/doi/10.1021/acsomega.5c10490.

Geometric configurations of GaAs/InSb interfaces (Figure S1); calculated interface formation energies (Table S1); charge density difference profiles and planar-averaged plots (Figure S2); projected density of states (PDOS) with spin–orbit coupling (Figure S3); evolution of EPR linewidth and amplitude as a function of radiation dose (Figure S4); EPR spectra of samples irradiated at 40 kGy (Figure S5); redshift of the Raman LO phonon mode vs dose (Figure S6); best-fit parameters of the EPR *g*-tensor (Table S2); detailed Raman peak parameters for MBE and MOCVD samples (Tables S3 and S4); experimental parameters for Raman measurements (Table S5); Raman laser power-dependence tests (Figure S7); and comparison with recent studies on radiation-induced defects (Table S6) (PDF)
