# Supplementary material for: Experimental and Theoretical Study of Defect Evolution in InSb Epilayers under Gamma Irradiation: A Comparative Analysis of MOCVD vs MBE Growth Methods
Source: ACS Omega. 2025 Dec 15;10(51):63474–87. doi: 10.1021/acsomega.5c10490 (PMC12756780; doi:10.1021/acsomega.5c10490)
Supplement: Supplementary file 1 [file ao5c10490_si_001.pdf]

Experimental and Theoretical Study of Defect Evolution in InSb Epilayers Under Gamma Irradiation: A comparative analysis of MOCVD vs. MBE Growth Methods

John Fredy Ricardo Marroquin[1], Alex Cortes Derc[1], Erika Nascimento Lima[2], Igor Saulo Santos de Oliveira[3], Mustafa Gunes[4], Mustafa Akyol[4], Braulio Archanjo[5], Walter Azevedo[6], Mohamed Henini[7], and Jorlandio Francisco Felix[1,*]

[1]*Institute of Physics, LabINS, University of Brasília (UnB), Brasília, DF 70910-900, Brazil*

[2]*Instituto de Física, Universidade Federal de Mato Grosso, 78060-900 Cuiabá, MT, Brazil*

[3]*Departamento de Física, Universidade Federal de Lavras, C.P. 3037, 37203-202 Lavras, MG, Brazil*

[4]*Department of Materials Science and Engineering, Adana Alparslan Türkeş Science and Technology University, TR 01250 Adana, Turkey*

[5]*Materials Metrology Division, National Institute of Metrology, Quality and Technology (INMETRO), Duque de Caxias, Rio de Janeiro 25250-020, Brazil*

[6]*Departamento de Química Fundamental, Universidade Federal de Pernambuco, 50740-560 Recife, PE, Brazil*

[7]*School of Physics and Astronomy, Nottingham Nanotechnology and Nanoscience Center, University of Nottingham, Nottingham NG7 2RD, UK*

E-mail: jorlandio@unb.br

DFT-Based Computational Setup

Prior to constructing the GaAs/InSb interface models, we independently optimized the lattice parameters of bulk GaAs and InSb. The resulting relaxed lattice constants, 5.76 Å for GaAs and 6.65 Å for InSb, are in close agreement with the experimental values of 5.6533 Å [1] and 6.479 Å,[1,2] respectively. Based on these results, the lattice mismatch between the two structures is approximately 15.45%, taking the GaAs bulk as the reference.

Simulations based on density functional theory (DFT) of interfaces with significant lattice mismatch can be computationally impractical. To tackle this issue for the GaAs/InSb system, we examined a lattice mismatch of 15.45 % , accommodating this strain on the GaAs side in our simulations. Although we recognize that applying such a level of strain to GaAs may lead to effects beyond the interface, our primary objective is to develop a qualitative model to investigate how zinc (Zn) doping on the InSb side of the GaAs/InSb interface influences the electronic states near the Fermi level.

The GaAs/InSb interface was modeled using supercells oriented in the *xy* plane, featuring a 1×1 lateral periodicity. The structure consists of a 4-monolayer (ML) InSb slab stacked on top of a 4-ML GaAs slab, with both slabs containing the same number of atoms. This configuration is aligned along the [001] crystallographic direction, as shown in Fig. S1. To eliminate spurious interactions between periodic images of the supercell, a 15 Å vacuum region is introduced along the same direction. In addition, to isolate and examine only the interfacial effects during structural relaxation, the two atomic layers closest to the vacuum region were kept fixed.

Interface Stability

To investigate the effects of Zn doping in an InSb slab at the GaAs/InSb interface, it is necessary first to identify the most stable stacking configuration. To achieve this, we evaluate

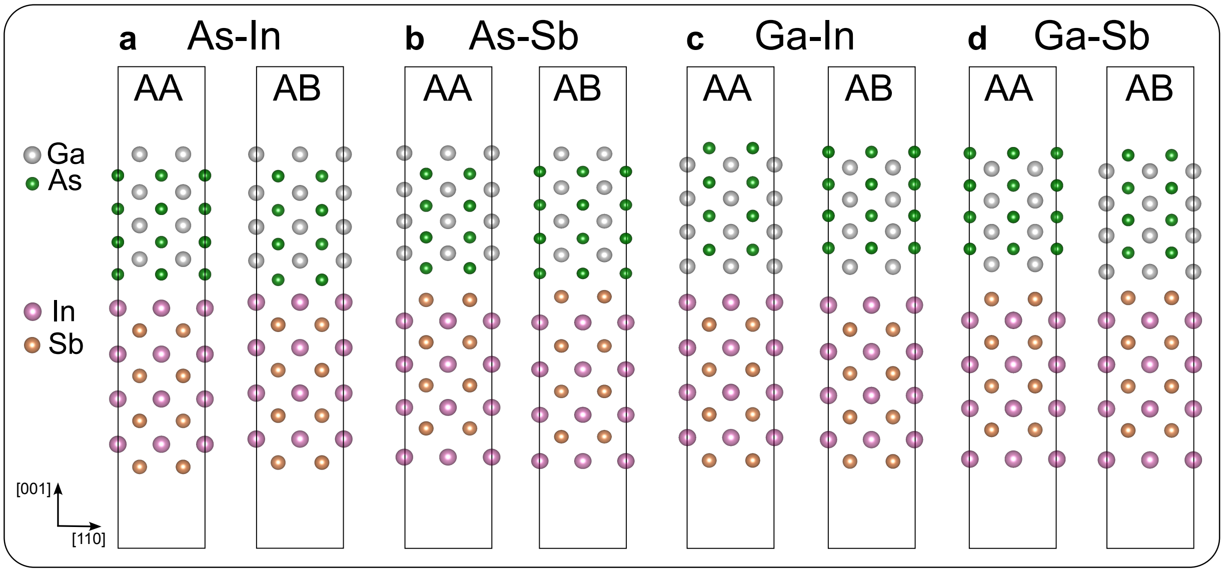

Figure S1: Side views of the four GaAs/InSb interface configurations considered in this study: (a) In-As, (b) As-Sb, (c) Ga-In, and (d) Ga-Sb. For each atomic arrangement, both AA and AB stacking types are shown, illustrating the lateral alignment between the GaAs and InSb layers at the interface.

the interface formation energy (E_F), defined by Equation 1:

$$E_F = E_{GaAs/InSb} - E_{GaAs} - E_{InSb}, \tag{1}$$

where $E_{GaAs/InSb}$ is the total energy of the GaAs/InSb interface configurations, E_{GaAs} and E_{InSb} correspond to the total energies of the individual GaAs and InSb slabs, respectively.

To this end, we analyze four distinct interfacial atomic arrangements: In–As [Fig. S1a], As–Sb [Fig. S1b], Ga–In [Fig. S1c], and Ga–Sb [Fig. S1d]. For each of these configurations, both AA and AB stacking types are considered, where AA stacking refers to a direct vertical alignment of atomic species across the interface, and AB stacking involves a lateral shift between the two slabs, resulting in an offset alignment of interfacial atoms. The corresponding formation energies for each case are summarized in Table S1, which shows that among the configurations examined, the In-As interface with the AB stacking is the most stable configuration.

Table S1: Interface formation energies (E_F) for the GaAs/InSb configurations considered in this study. The first column indicates the interfacial atomic arrangement. E_F(AA) and E_F(AB) represent the formation energies corresponding to the AA and AB stacking types, respectively, calculated according to Equation 1. All values are given in (eV).

Interfacial Configurations	E_F(AA)	E_F(AB)
As-In	-116.69824310	-116.93533138
As-Sb	-110.79365053	-114.97124633
Ga-In	-115.88983023	-115.12140446
Ga-Sb	-116.79806697	-116.34020233

Differential charge density and Bader charge analysis

To further characterize the interfacial interactions, we evaluated the charge transfer (CT) between InSb and the GaAs substrate using the Bader charge analysis method.[3,4] The pristine interface exhibits a net transfer of $CT = 1.50 \times 10^{14}$ e/cm^2 from InSb to GaAs. Upon Zn incorporation on the InSb side, this value increases slightly to $CT = 1.54 \times 10^{14}$ e/cm^2, indicating that doping only modestly enhances the electron donation toward the GaAs substrate. To gain spatially resolved insights into the charge redistribution, we computed the charge density difference, defined as

$$\Delta\rho(\mathbf{r}) = \rho_{\mathrm{InSb/GaAs}}(\mathbf{r}) - \rho_{\mathrm{InSb}}(\mathbf{r}) - \rho_{\mathrm{GaAs}}(\mathbf{r}), \tag{2}$$

where $\rho_{\mathrm{InSb/GaAs}}(\mathbf{r})$ is the charge density of the full interface, while $\rho_{\mathrm{InSb}}(\mathbf{r})$ and $\rho_{\mathrm{GaAs}}(\mathbf{r})$ correspond to the isolated InSb and GaAs slabs, respectively. For a more quantitative representation along the direction normal to the interface, we also evaluated the planar-averaged profile,

$$\Delta\rho(z) = \frac{1}{A_{xy}} \int_{A_{xy}} \Delta\rho(\mathbf{r})\, dx\, dy, \tag{3}$$

where A_{xy} denotes the supercell area parallel to the interface.

The results are summarized in Fig. S2. The background isosurface plots display the three-dimensional distribution of $\Delta\rho(\mathbf{r})$, while the superimposed curves show the planar-

averaged $\Delta\rho(z)$. In Fig. S2(a), corresponding to the pristine InSb/GaAs interface, regions of charge accumulation (GaAs side) and depletion (InSb side) are clearly visible, reflecting the interfacial polarization. Figure S2(b) presents the Zn-doped system, which exhibits a similar redistribution pattern but with slightly enhanced electron transfer toward GaAs. In both cases, the most pronounced peaks and valleys are confined within the first few atomic layers, whereas the inner layers remain nearly neutral. This localization of electronic reconstruction, together with the modest increase in CT upon doping, confirms that Zn incorporation subtly reinforces the interfacial charge transfer without significantly altering the overall distribution.

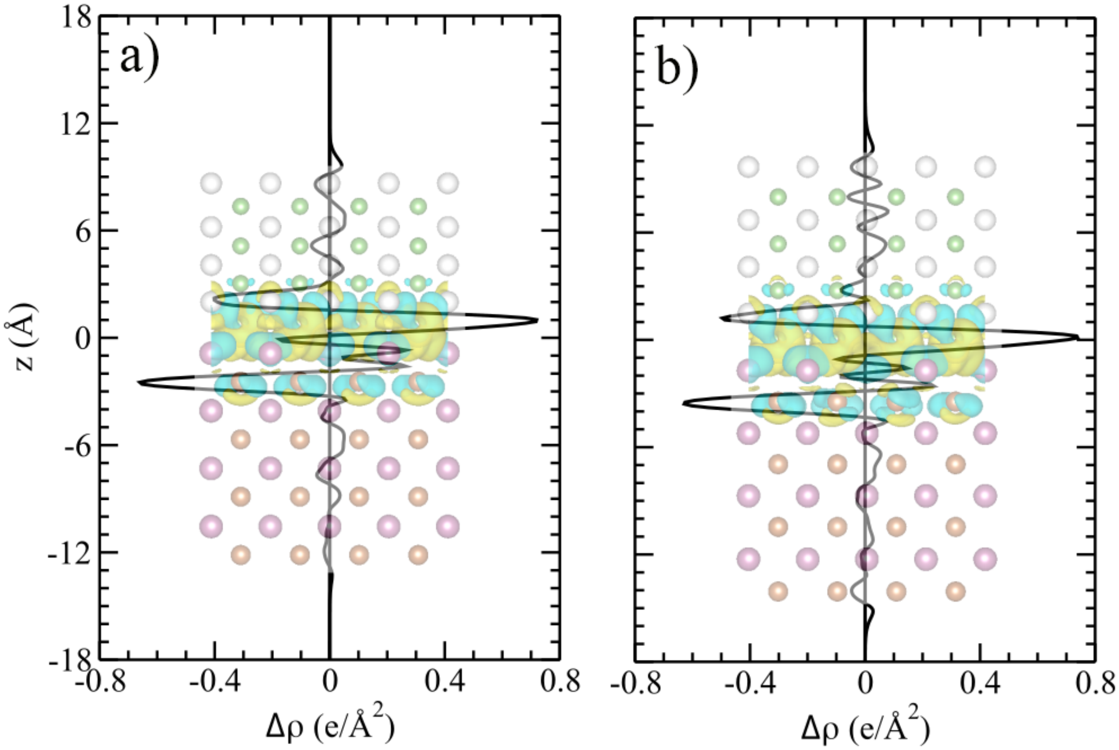

Figure S2: Charge density difference $\Delta\rho(\mathbf{r})$ (background isosurfaces) and planar-averaged profiles $\Delta\rho(z)$ for (a) the pristine InSb/GaAs interface and (b) the Zn-doped InSb/GaAs interface. Yellow and cyan regions denote electron accumulation and depletion, respectively.

Spin–Orbit Coupling Analysis

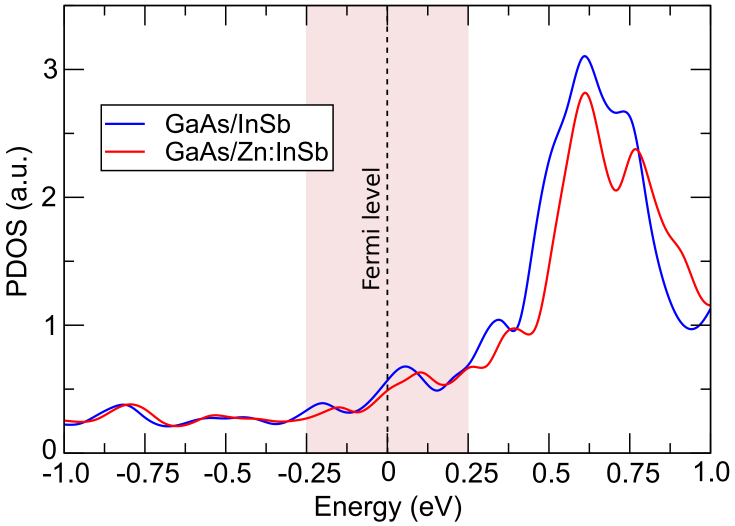

Figure S3: Projected density of states (PDOS) for the pristine GaAs/InSb (InSb-MB) and Zn-doped GaAs/InSb (InSb-MO) interfaces including spin-orbit coupling. The main peak near the valence-band edge (around -0.2 eV) remains essentially unchanged, while the InSb-MO sample exhibits a lower PDOS at the Fermi level and a less pronounced conduction-band peak compared to InSb-MB, confirming the improved interfacial electronic quality upon Zn incorporation.

Relation of Peak-to-Peak amplitude and linewidth with Radiation Dose

Figure S4 show the behavior of the linewidth (ΔH) and the signal amplitude (ΔA) for both samples InSb-MO and InSb-MB as function of radiation dose. Figure S4a illustrates the linewidth behavior for the InSb-MO sample as a function of radiation dose. Initially, the linewidth broadens for doses up to 10 kGy, a trend attributed to the generation of defects and increased structural disorder by the radiation. Conversely, at doses above 10 kGy, the linewidth begins to narrow. For InSb-MB sample the linewidth broadens for doses up to 1 kGy. With increasing dose, the linewidth decreases drastically up to 5 kGy and continues to decrease slowly thereafter.

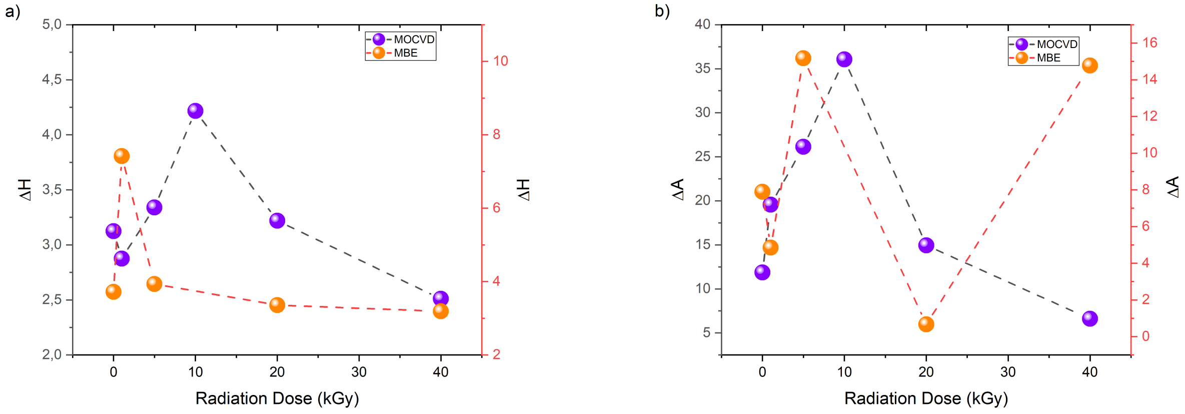

Figure S4: Relationship of (a) linewidth and (b) peak-to-peak amplitude to different radiation doses for both InSb-MO and InSb-MB.

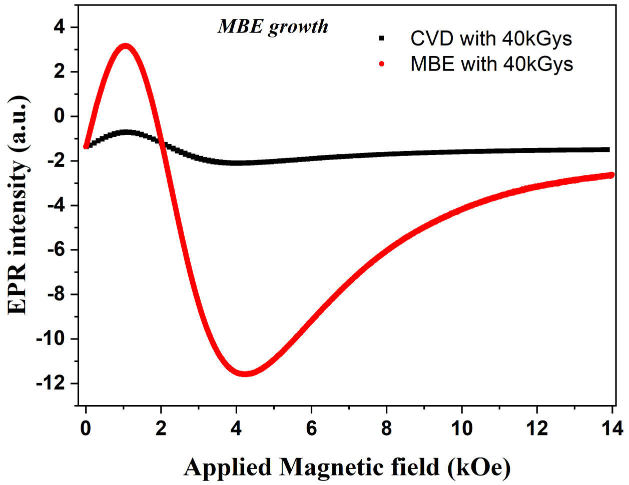

Figure S5: EPR spectrum of InSb-Mo and InSb-MO irradiated with gamma radiation using a dose of 40 kGy.

Figure S4b shows the amplitude of the EPR signal of both samples. For InSb-MO sample the amplitude increases from ∼19 to a peak of ∼36 at 10 kGy, and then drops drastically at higher doses. For the InSb-MB sample the behaviour is more complex, alternating between increases and decreases.

Figure S5 shows that the both samples are highly susceptible to high radiation dose (40kGy). For InSb-MB induces a transition to a phase with strong magnetic ordering, whereas the InSb-MO sample the signal is significantly vanished, exhibiting a different mag-

netic response under the same conditions.

Effects of Gamma Irradiation on Raman Spectroscopy of InSb Samples

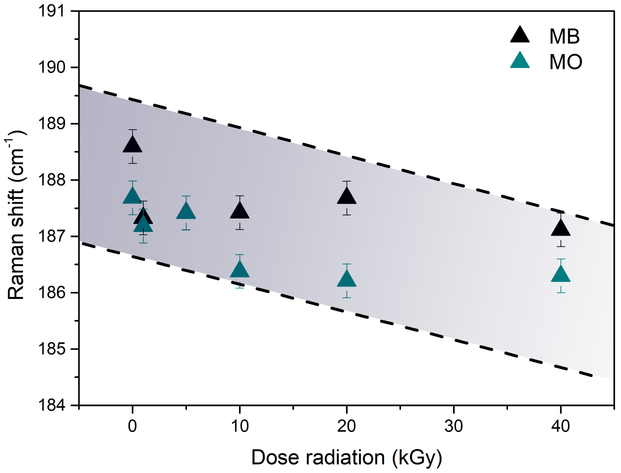

Figure S6: The redshift of the LO phonon mode's center frequency for both samples as a function of radiation dose, indicating lattice softening and increased strain. Diagrams of the InSb lattice.

Raman spectroscopy provides additional evidence for radiation-induced defect formation, as presented in Figure S6. Specifically, a redshift is observed in the LO phonon mode for both the InSb-MB and InSb-MO samples. This phenomenon is a direct consequence of lattice relaxation caused by defects. These structural imperfections disrupt the periodic potential of the crystal, dampening the atomic vibrational modes and lowering their characteristic frequency, which manifests as the observed redshift.

Table S2: Best-fit parameters of the axially symmetric g-tensor, $g_{\mathrm{res}}(\theta) = \sqrt{g_\perp^2 \sin^2\theta + g_\parallel^2 \cos^2\theta}$, obtained from non-linear least-squares fits to the angular dependence of the EPR resonance field for InSb-MB and InSb-MO at room temperature. Uncertainties correspond to 95% confidence intervals (CIs). RMS is the root-mean-square fit residual.

Sample	$g_\perp$	$g_\parallel$	$\Delta g = g_\perp - g_\parallel$	95% CI for $g_\perp$	95% CI for $g_\parallel$	95% CI for Δg
InSb-MB	20.82	20.15	0.67	[20.62, 21.02]	[19.95, 20.35]	[0.61, 0.73]
InSb-MO	27.48	27.29	0.19	[27.30, 27.66]	[27.11, 27.47]	[0.15, 0.23]

Table S3: Raman peak parameters for InSb MBE samples as a function of γ-dose.

Dose (kGy)	Amplitude	Center (cm^{-1})	FWHM (cm^{-1})	err(Amp)	err(Center)	err(FWHM)	Atribution	Reference
**Peak around 187 cm^{-1} (MBE)**								
0	0.05124	188.790	6.81	0.10605	0.0397	0.412	TO	[?]
1	0.02217	187.326	9.72	0.02172	0.1374	0.237	TO	
5	0.02870	187.418	9.21	0.03380	0.1147	0.186	TO	
10	0.01627	187.422	9.11	0.01563	0.1615	0.267	TO	
20	0.02901	187.680	7.63	0.06160	0.0777	0.474	TO	
40	0.02265	187.115	9.49	0.01843	0.1609	0.201	TO	
**Peak around 180 cm^{-1} (MBE) – LO branch**								
0	0.00979	183.241	16.37	0.08703	2.0012	2.387	LO	[?]
1	0.00908	180.990	12.47	0.02195	1.0467	1.358	LO	
5	0.00571	181.143	11.39	0.01771	1.2862	1.818	LO	
10	0.00771	180.168	13.45	0.01846	1.0992	1.567	LO	
20	0.00996	183.796	12.76	0.04151	1.0023	1.023	LO	
40	0.01047	180.545	11.92	0.01995	0.8118	1.094	LO	
**Peak around 140 cm^{-1} (MBE)**								
0	–	–	–	–	–	–	–	–
1	0.01974	142.823	11.45	0.00912	0.0391	0.135	Sb-Sb mode	[?]
5	0.01490	145.418	11.82	0.01673	0.0592	0.254	Sb-Sb mode	
10	0.02906	140.946	11.91	0.00803	0.0265	0.092	Sb-Sb mode	
20	0.00688	146.395	11.72	0.00507	0.1554	0.492	Sb-Sb mode	
40	0.02132	142.030	11.89	0.00700	0.0361	0.123	Sb-Sb mode	

Table S4: Raman peak parameters for InSb MOCVD samples as a function of γ-dose.

Dose (kGy)	Amplitude	Center (cm^{-1})	FWHM (cm^{-1})	err(Amp)	err(Center)	err(FWHM)	Atribution	Reference
**Peak around 187 cm^{-1} (MOCVD) – TO**								
0	0.03991	187.685	8.51	0.05693	0.105	0.170	TO	?
1	0.02157	187.181	9.55	0.32837	0.181	0.223	TO	
5	0.02764	187.414	8.30	0.37820	0.124	0.217	TO	
10	0.00175	186.376	5.20	0.01369	0.415	1.274	TO	
20	0.00530	186.211	8.80	0.06505	0.257	0.702	TO	
40	0.00719	186.298	9.11	0.09016	0.274	0.706	TO	
**Peak around 180 cm^{-1} (MOCVD) – LO**								
0	0.00985	182.298	10.14	0.13087	0.693	0.878	LO	?
1	0.00664	179.545	11.43	0.08088	0.781	1.290	LO	
5	0.00781	180.882	11.10	0.09862	0.833	1.288	LO	
10	0.00083	177.363	6.74	0.00597	0.799	2.299	LO	
20	0.00314	176.414	7.50	0.02514	0.303	0.762	LO	
40	0.00301	176.923	8.22	0.02639	0.490	1.174	LO	
**Peak around 140 cm^{-1} (MOCVD) – Peak 3**								
0	–	–	–	–	–	–	–	–
1	0.02146	142.819	10.44	0.042	0.536	0.465	Sb-Sb mode	?
5	0.02039	145.720	10.68	0.018	0.143	0.329	Sb-Sb mode	
10	0.04747	140.391	10.34	0.010	0.041	0.094	Sb-Sb mode	
20	0.04123	138.808	11.04	0.015	0.109	0.171	Sb-Sb mode	
40	0.03571	139.262	12.21	0.019	0.184	0.281	Sb-Sb mode	
**Peak 2 (MOCVD) – low-frequency shoulder around 132–139 cm^{-1}**								
0	–	–	–	–	–	–	–	–
1	0.00349	139.249	6.78	0.040	1.474	2.570	Amorphous mode	?
5	0.00354	139.204	5.89	1.177	0.384	1.797	Amorphous mode	
10	0.00490	132.176	8.06	0.018	0.310	0.788	Amorphous mode	
20	0.00464	131.632	9.17	0.023	0.683	1.206	Amorphous mode	
40	0.00343	131.909	8.60	0.020	0.979	1.937	Amorphous mode	

Table S5: The specific parameters used for Raman spectroscopy measurements.

**Parameter**	**Value (Post-Objective)**
Applied Power (Max)	25 mW
Objective/NA	$100\times$ /NA = 0.90
Spot Size	$\approx 0.5\ \mu$m
Power Density (Max)	$\approx 12.7\ \mathrm{MW}\cdot\mathrm{cm}^{-2}$

The Raman *power-dependence test* performed at laser powers of 25 mW, 40 mW, and 50 mW. Figure S7(a) shows the normalized Raman spectra for the three excitation powers, confirming that the overall line shape remains unchanged. Figure S7(b) and Figure S7(c) display the extracted Raman peak position and the full width at half maximum (FWHM) of the TO mode, respectively. Both quantities exhibit only negligible variation (within experimental uncertainty), indicating that no laser-induced redshift or power-dependent broadening occurs. These results confirm that the Raman shifts and linewidth changes discussed in the main text originate from *intrinsic structural modifications* in the irradiated InSb, rather

than from laser heating or other excitation-power artifacts.

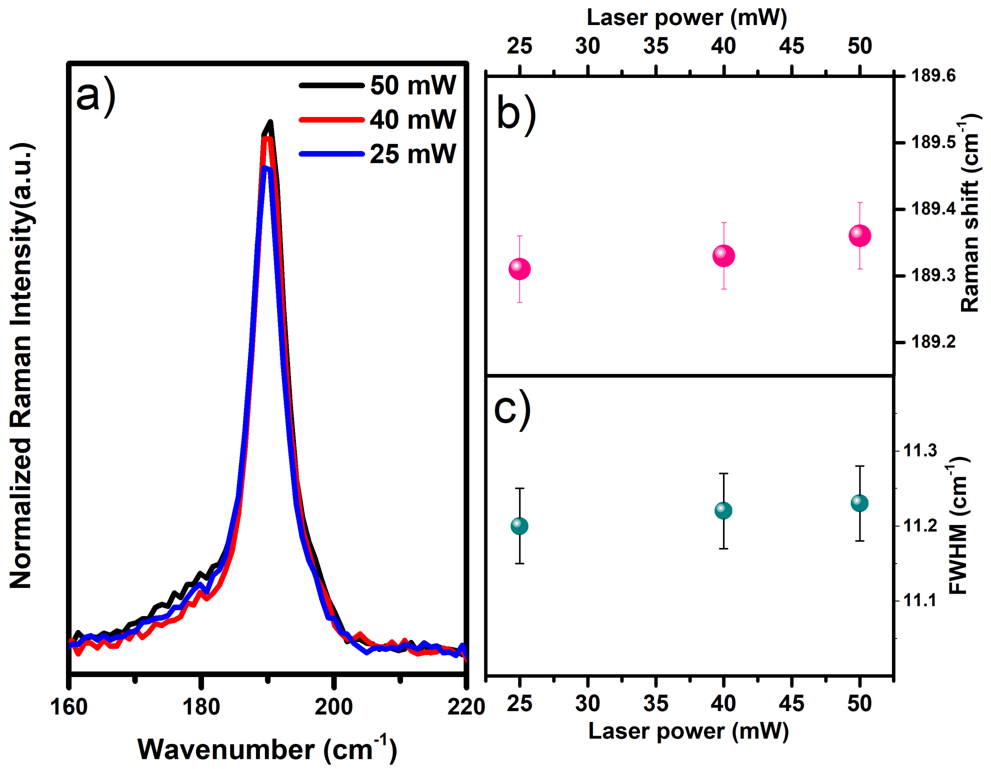

Figure S7: Raman power-dependence test for InSb. (a) Normalized Raman spectra acquired at excitation powers of 25 mW, 40 mW, and 50 mW, showing no observable changes in line shape. (b) Extracted Raman peak position for the three excitation powers, demonstrating negligible variation within experimental uncertainty.(c) Full width at half maximum (FWHM) of the TO mode as a function of laser power, also displaying minimal deviation.

Table S6: Comparison between the present work and recent studies on radiation-induced defects in InSb and related semiconductor systems (Part 1/2).

**Study**	**Material / structure**	**Radiation conditions**	**Main techniques**	**Main findings and relation to this work**
This work	InSb epilayers (1 μm) on GaAs(001) grown by MBE (InSb-MB) and MOCVD with a Zn-doped seed layer (InSb-MO)	^{60}Co γ-rays, dose rate $\approx$ 2.7 kGy h^{-1}, cumulative doses 0–40 kGy at room temperature	EPR (X-band), Raman, XRD, HRSTEM/HAADF, DFT	MOCVD sample shows superior initial crystallinity (Zn:InSb interface passivation), but under high γ doses it becomes more susceptible to electronic degradation and stoichiometry violations. MBE sample, with higher initial dislocation density, exhibits defect saturation and partial recovery of the EPR signal at high doses. Establishes a growth-method-dependent trade-off between initial crystalline quality and long-term radiation "survivability", and proposes EPR g-factor anisotropy and Raman FWHM as non-destructive RHA metrics.
Ďuran et al. (2011) [5]	InSb thin-film Hall plates designed for ITER ex-vessel steady-state magnetic diagnostics	Reactor neutron irradiation up to fluences of order 10^{17} n cm^{-2} with post-irradiation temperature cycling	Hall-effect measurements (carrier density, mobility, sensitivity)	Demonstrates that highly Sn-doped InSb sensors retain Hall sensitivity within a few percent under ITER-relevant neutron fluences, emphasizing the role of high initial carrier concentration for radiation hardness. Relates to the present work by showing macroscopic electrical stability, while our study provides a microscopic picture of defect generation and self-healing under ionizing (γ) radiation.
Jankowski et al. (2019) [6]	InSb-based Hall sensors for neutron-resistant operation	Neutron irradiation in fusion-reactor-relevant environments (fluences approaching 10^{17}–10^{18} n cm^{-2})	Hall-effect characterization (sensitivity, offset, noise) and sensor design optimization	Optimizes geometry and doping to obtain InSb Hall sensors with stable performance under high neutron fluence, but does not address the microscopic defect landscape. Our work complements this by correlating growth method, interfacial chemistry and spectroscopic signatures (EPR, Raman) with the radiation response of InSb epilayers.
El-Ahmar et al. (2022) [7]	InSb-based thin films on SiC and graphene/SiC Hall sensors for extreme magnetic diagnostics	Neutron irradiation and elevated temperatures relevant to ITER-like conditions	Hall-effect measurements vs. fluence and temperature	Compares InSb thin films with graphene on SiC, showing that InSb devices suffer measurable degradation in sensitivity and offset under neutron irradiation, while graphene-based sensors remain more robust. The present work extends this line of research by providing a detailed spectroscopic and structural analysis of radiation-induced defects in InSb, clarifying the microscopic mechanisms behind such macroscopic degradation.

Table S6: (Continued) Comparison between the present work and recent studies on radiation-induced defects.

**Study**	**Material / structure**	**Radiation conditions**	**Main techniques**	**Main findings and relation to this work**
Reddig et al. (2024) [8]	Thin-film and two-dimensional magnetic field sensors (including InSb-based platforms and graphene)	Neutron radiation in harsh fusion-relevant environments	Electrical sensor characterization under neutron exposure	Reports comparative neutron-radiation effects in thin-film vs. 2D magnetic sensors, focusing on device-level performance metrics rather than atomic-scale defects. In contrast, our study delivers a multi-modal defect analysis for InSb epilayers under γ irradiation, which is directly useful for correlating macroscopic sensor stability with microscopic defect evolution.
Duinong et al. (2022) [9]	ZnO and Mg-doped ZnO thin films (300 nm) on ITO substrates	^{60}Co γ-rays, total ionizing dose 10–300 kGy at 2 kGy h^{-1}	XRD, AFM, UV–Vis spectroscopy, Monte Carlo simulation	Shows that increasing γ dose leads to increased lattice strain, reduced crystallite size, surface roughening and band gap narrowing due to colour-centre formation. These trends are consistent with our observation that γ irradiation in InSb induces point defects and local structural disorder, reflected in Raman-band broadening and changes in the EPR linewidth, even though InSb is a narrow-gap III–V while ZnO is a wide-gap oxide.
Lee et al. (2018) [10]	InAs/GaSb superlattices containing InSb-like interfacial layers for infrared detectors	γ-ray exposure at space-relevant doses combined with temperature variation	Electrical transport measurements and device modelling	Finds that γ irradiation predominantly introduces ionization-related defects that degrade carrier transport without catastrophic structural amorphization, highlighting the importance of interface quality in narrow-bandgap heterostructures. Our work reaches similar conclusions for InSb/GaAs: the Zn-doped interface initially improves crystallinity but becomes a weak point for radiation-induced electronic degradation at high γ doses.

References

(1) Madelung, O., Rössler, U., Schulz, M., Eds. *Landolt-Börnstein: Numerical Data and Functional Relationships in Science and Technology – Group III Condensed Matter, Vol. 41B: Semiconductors*; Springer-Verlag: Berlin Heidelberg, 2001.

(2) Lide, D. R., Ed. *CRC Handbook of Chemistry and Physics*, 102nd ed.; CRC Press: Boca Raton, FL, 2021.

(3) Bader, R. F. W. *Atoms in Molecules - A Quantum Theory*; Oxford University Press: Oxford, 1990.

(4) Henkelman, G.; Arnaldsson, A.; Jónsson, H. A fast and robust algorithm for Bader decomposition of charge density. *Comput. Mater. Sci.* **2006**, *36*, 354–360.

(5) Duran, I.; Entler, S.; Grover, O.; Bolshakova, I.; Vyborny, K.; Kocan, M.; Jirman, T.; Vayakis, G. Steady-state magnetic diagnostic for ITER and beyond. Proceedings of the 30th Symposium on Fusion Technology (SOFT-30). 2018; P4.061.

(6) Jankowski, J.; Prokopowicz, R.; Pytel, K.; El-Ahmar, S. Toward the Development of an InSb-Based Neutron-Resistant Hall Sensor. *IEEE Transactions on Nuclear Science* **2019**, *66*, 926–931.

(7) El-Ahmar, S.; Jankowski, J.; Czaja, P.; Reddig, W.; Przychodnia, M.; Raczyński, J.; Koczorowski, W. Hall-effect sensors for extreme temperature applications. 2024; https://nano.put.poznan.pl/wp-content/uploads/2024/09/EuroSensors2024_SA.pdf, Oral presentation.

(8) Reddig, W.; El-Ahmar, S.; Prokopowicz, R.; Ciuk, T. Neutron Radiation Effects on Thin-Film and Two-Dimensional Magnetic Field Sensors. 2024; https://nano.put.poznan.pl/wp-content/uploads/2024/09/EuroSensors2024_WR.pdf, Oral presentation.

(9) Duinong, M.; Rasmidi, R.; Chee, F. P.; Moh, P. Y.; Salleh, S.; Mohd Salleh, K. A.; Ibrahim, S. Effect of Gamma Radiation on Structural and Optical Properties of ZnO and Mg-Doped ZnO Films Paired with Monte Carlo Simulation. *Coatings* **2022**, *12*, 1590.

(10) Lee, J.; Fredricksen, C. J.; Flitsiyan, E.; Peale, R. E.; Chernyak, L.; Taghipour, Z.; Casias, L.; Kazemi, A.; Krishna, S.; Myers, S. Impact of temperature and gamma radiation on electron diffusion length and mobility in p-type InAs/GaSb superlattices. *Journal of Applied Physics* **2018**, *123*, 235104.
